# Intracavernous Injection of Mechanically Extracted Stromal Vascular Fragments Suppresses Endothelial‐Mesenchymal Transformation to Mitigate Erectile Dysfunction in Hypertensive Rats

**DOI:** 10.1155/sci/3343152

**Published:** 2026-01-16

**Authors:** Cheng Shao, Yi Sun, Jun Zhao, Chao Ju, Tianli Yang, Jingyu Liu, Liuhua Zhou, Ruipeng Jia, Feng Zhao

**Affiliations:** ^1^ Department of Urology, Nanjing First Hospital, Nanjing Medical University, Nanjing, China, njmu.edu.cn

**Keywords:** cellular stromal vascular fraction, endothelial-mesenchymal transformation, erectile dysfunction, hypertension, stem cell, tissue adipose stromal vascular fraction

## Abstract

Erectile dysfunction (ED) is widespread among individuals with high blood pressure and negatively affects quality of life. The effect of stromal vascular fraction (SVF) on hypertension‐related ED remains unexplored. We used a hypertensive rat model to explore the relative efficacy of adipose tissue stromal vascular fraction (tSVF) and cellular SVF (cSVF). We then investigated the possible mechanisms of these treatments. Hypertensive rats were divided into three groups according to different treatments. Their intracavernous pressure (ICP) during erection and condition of cavernous tissue were compared to those of the controlled group. Endothelial‐mesenchymal transformation (EndMT) markers as well as related inflammatory factors were also measured. cSVF and tSVF were labeled with CM‐Dil before injection in order to determine whether cSVF and tSVF survived, proliferated, and transdifferentiated in vivo. The increased ICP during erection demonstrated that tSVF treatment significantly improved hypertension‐related ED. tSVF increased the smooth muscle‐to‐collagen ratio and inhibiting the expression of fibrosis‐related proteins in hypertensive rats while rescuing the expression of vWF and eNOS, which indicated the preserving of endothelial tissue of the penis. Immunofluorescence staining and western blotting of penile tissue clearly suggest the inhibitory effect of tSVF on the overoccurring EndMT. Immunofluorescence staining and Western blot analysis of endothelial cells in vitro corroborate the whole‐tissue findings. The experiments in N‐nitro‐L‐arginine methyl ester hydrochloride (L‐NAME)–induced human umbilical vein endothelial cells (HUVECs) revealed tSVF suppresses EndMT via inhibiting the TGF‐*β*2–Smad2/Smad3 pathway. In vivo tSVF and cSVF tracing suggested that tSVF showed better longevity and transdifferentiation capacity than cSVF, thus exerting a more significant therapeutic effect. Treatment with tSVF significantly reserved erectile function in a hypertensive rat model. The mechanism appears to be inhibition of pathological EndMT through self‐differentiation. We conclude that tSVF is a promising therapeutic candidate for treating hypertensive ED.

## 1. Introduction

Erectile dysfunction (ED) is the persistent inability to attain and maintain an erection sufficient for satisfactory sexual performance [1]. American and British observational studies estimate the prevalence of complete ED as about 5% among 40‐year‐old men, 10% among men in their 60s, 15% among men in their 70s, and 30%–40% among men in their 80s [2]. Hypertension is strongly associated with ED, with prevalence among hypertensive patients exhibiting approximately double the prevalence as a normotensive population [3, 4]. The common pathogenesis of ED and hypertension is endothelial dysfunction that leads to abnormal vasoreactivity favoring vasoconstriction [5]. Furthermore, some antihypertensive drugs (e.g., thiazide diuretics, nonvasodilating beta‐blockers, and mineralocorticoid receptor antagonists) exacerbate ED [6]. Treatments of hypertension should therefore take sexual health into account.

The stromal vascular fraction (SVF) is a heterogeneous cell population which is the remaining ingredients after removing mature fat cells in the adipose tissue of transplantation. They comprise endothelial cells, smooth muscle cells, blood cells, and mesenchymal cells [7]. Given their capacity to differentiate into various mesodermal lineages and secrete growth factors, SVF possesses ideal characteristics for use in cell therapy and tissue engineering [8, 9]. Depending on whether collagenase is used during extraction, SVF is divided into cellular SVF (cSVF) and tissue SVF (tSVF) [10]. While cSVF extraction results in complete disruption of cell attachment to the extracellular matrix (ECM), tSVF is a mixture of cellular debris, destroyed blood cells, and ECM fragments [11]. Thus, tSVF retains native ECM and perivascular structures, limiting death caused by cellular “loss of nest.” In turn, the biological activity of transplanted cells is higher than that of cSVF [12, 13].

Treatment with cSVF successfully mitigated ED in rat models of cavernous nerve injury [14, 15]. Increases in smooth muscle/collagen ratio and von Willebrand factor (vWF) expression were higher in the cSVF group than in the adipose‐derived stem cell (ADSC)–treated group [16]. However, the effect of cSVF and tSVF on hypertension‐related ED remains unexplored.

The endothelial‐mesenchymal transition (EndMT) occurs when an endothelial cell phenotype switches to a mesenchymal cell (e.g., myofibroblasts and smooth muscle cells). Growing evidence suggests that excessive EndMT has a strong relationship with cardiovascular disease and is implicated in pulmonary arterial hypertension, atherosclerosis, fibrosis, and cancer [17–20]. Specifically, hypertension‐related endothelial cell damage and fibrosis are closely associated with EndMT because the transition involves inflammatory cytokines TGF‐*β*2, IL‐1*β*, and TNF‐*α* [21–23]. Inhibiting EndMT improves erectile function in a diabetic rat model [24]. However, a similar study has not yet been performed in a hypertensive rat model; therefore, we do not know whether the results are broadly applicable.

Hence, in this study, we established a hypertensive rat model to explore whether tSVF is an appropriate treatment for ED. We compared its effects with those of cSVF and determined the underlying mechanisms. We performed labeling experiments with CM‐Dil to better visualize tSVF biological behavior in the cavernous body. Our findings can contribute to the discovery of novel therapies for hypertensive ED.

## 2. Materials and Methods

### 2.1. Extraction and Characterization of cSVF and tSVF

cSVF was isolated following our previous protocol [25]. After anesthetization, ~5 mL of epididymal fat was removed from the rats and minced with ophthalmic scissors. Minced fat was poured into DMEM containing 0.075% collagenase type I and digested at 37°C for 1 h. After centrifugation, filtration, and erythrocyte lysis, the final product was resuspended in DMEM containing fetal bovine serum to yield a cSVF suspension. Minced fat was first placed into a 20 mL syringe and connected to another 20 mL syringe via a converter (aperture size = 2.4 mm). The syringes were then depressed at a consistent speed, pushing the fat back and forth 30 times through the converter. Subsequently, converters with successively smaller aperture sizes (1.0 mm, 0.6 mm, and 0.2 mm) were switched in. The fat was agitated again 30 times per converter. Centrifugation then separated the fat samples into three layers; the middle layer was tSVF. Cell markers of uncultured SVF were explored via flow cytometry using the following antibodies: fluorescein isothiocyanate (FITC)–conjugated anti‐CD45 (BioLegend), FITC–conjugated anti‐CD90 (Bioss), phycoerythrin (PE)–conjugated anti‐VEGFR2 (Cell Signaling), PE–conjugated anti‐CD106 (Bioss), PE–conjugated anti‐CD31 (ThermoFisher), PE–conjugated anti‐CD29 (ThermoFisher), and allophycocyanin (APC)–conjugated anti‐CD11b/c (BD Biosciences). Labeled SVF was analyzed using a FACSCalibur instrument (BD Biosciences). An isotype‐matched IgG was used for each procedure.

### 2.2. CM‐Dil Labeling of cSVF and tSVF

cSVF and tSVF were labeled with CM‐Dil before transplantation following the manufacturer’s protocol. Briefly, both fractions were incubated in CM‐Dil (1 μg/mL) for 5 min at 37 °C and then for 15 min at 4°C. Labeled cSVF or tSVF was injected into the corpora cavernosa and detected using fluorescence microscopy (Zeiss) after 1, 2, and 4 weeks.

### 2.3. Animal Experiments

The study was approved by the Ethics Committee for the Use of Experimental Animals of the Nanjing First Hospital, Nanjing Medical University. Male Sprague–Dawley (SD) rats were 8–12 weeks old and housed at the Experimental Animal Center of Nanjing First Hospital. The food and water were abundant and can be obtained by rats easily. All studies were conducted under the allowance of the Guidelines for the Care and Use of Laboratory Animals of the National Institutes of Health (NIH).

Hypertension in rats was induced using N‐nitro‐L‐arginine methyl ester hydrochloride (L‐NAME). L‐NAME is the most prominent of L‐arginine analogs, widely used inhibitors of nitric oxide synthase (NOS) in vitro and in vivo [26]. Chronic L‐NAME administration can induce hypertensive ED that is irreversible with ED drug sildenafil [27].

For 6 weeks, normal (control) rats were provided purified water daily, while the hypertensive rats were administered L‐NAME (40 mg/kg, p.o.) at 1 mL/100 g based on individual weight. At 2 weeks of administration, 0.2 mL of phosphate‐buffered saline (PBS), cSVF, and tSVF were injected into the corpora cavernosa using a BD U‐40 insulin syringe. Subjects were then divided into four groups based on variable treatments within groups. Noninvasive measurements of tail blood pressure, systolic blood pressure (SBP), diastolic blood pressure (DBP), and mean arterial pressure (MAP) were measured in all SD rats at 6 weeks of administration.

### 2.4. Noninvasive BP Measurement

The BP‐2010 AUL noninvasive BP monitoring system (Softron Biotechnology, Beijing, China) was used, as described previously [28]. Briefly, instrument air tightness was first examined, and incubator temperature was adjusted to 37 °C. The entire system was kept at a room temperature of 26 °C. Rats were placed in the monitoring environment for 15 min, with sensors attached around the tail root. SBP, DBP, and MAP were measured when the signal stabilized.

### 2.5. Evaluation of Erectile Function

At the 4th week postinjection, SD rats were anesthetized with 2.5%–3% isoflurane before exposing the pelvic ganglia and corpora cavernosum of the penis. A probe needle connected to a baroreceptor was inserted into the cavernous body and perfused with heparin saline (200 U/mL) to prevent blood reflux in the penis from blocking the needle. Next, the pelvic ganglion cavernous nerve branch was stimulated for 1 min with bipolar electrodes at settings of 25 Hz, 5 V, and 2 ms. Changes in intracavernous pressure (ICP) were recorded. In combination with the previously measured MAP, erectile function was evaluated using the ratios of ICPmax to MAP and total ICP to MAP.

### 2.6. Cell Culture and Treatment

Human umbilical vein endothelial cells (HUVECs) were purchased from the American Type Culture Collection (USA) and cultured in endothelial growth medium (ScienCell, USA) supplemented with 5% FBS (Gibco, USA) and endothelial growth factors. The confluent HUVECs at Passages 6–7 were maintained in basal medium containing 0.5% FBS for 8 h, followed by stimulation with L‐NAME (1 mM) for 72 h to induce EndMT or 1 mM L‐arginine treatment as a negative control [29, 30]. After 24 h of culture, cSVF and tSVF were introduced into transwell chambers for 48 h coculture with HUVECs. Thereafter, the cells were collected for further experiments.

### 2.7. Immunohistochemical Staining

Masson’s trichrome staining can distinguish between smooth muscle tissue (red) and fibrous tissue (blue); the ratio is an indicator of tissue fibrosis severity. Penis tissue samples were fixed in 4% paraformaldehyde and embedded in paraffin before sectioning (5 μm). Slices were stained with Masson three‐color for fibrotic assessment of the corpora cavernosum. After examining all sections under a microscope, three sections per rat were selected for statistical analysis. Collagen tissues (blue) and smooth muscles (red) were compared in ImageJ (NIH). Paraffin sections were immunostained with primary antibody against matrix metalloproteinase 2 (MMP2, 10373‐2‐AP, 1:100, Proteintech) and matrix metalloproteinase 9 (MMP9, 10375‐2‐AP, 1:100, Proteintech) and then counterstained with 4′,6‐diamidino‐2‐phenylindole (DAPI, Vector Laboratories) to label the nuclei. Images were captured by a fluorescence microscope (Zeiss) and analyzed by ImageJ (NIH).

### 2.8. Immunofluorescence Staining

Any remaining penile tissues were fixed, sectioned, permeabilized with 0.5% Triton X‐100, and blocked with 3% bovine serum albumin (BSA) for 1 h at room temperature. Sections were then incubated with antibodies against PCNA (sc‐71858, 1:100, Santa Cruz), eNOS (27120‐1‐AP, 1:50, Proteintech), SMA (ab7817, 1:100, Abcam), vWF (27186‐1‐AP, 1:100, Proteintech), IL‐1*β* (16806‐1‐AP, 1:100, Proteintech), TGF‐*β*2 (sc‐374658, 1:100, Santa Cruz), and TNF‐*α* (17590‐1‐AP, 1:100, Proteintech). After washing three times with PBS, sections were incubated with secondary antibodies in the dark for 1 h. Nuclei were stained with DAPI for 10 min and sealed with antifluorescence quenching blocker. For HUVECs, the cells with various treatments were fixed with 4% paraformaldehyde, infiltrated with 1% Triton X‐100, and sealed with 5% BSA. Subsequently, the aortic sections or HUVECs were incubated with primary antibodies against VE‐cadherin (27956‐1‐AP, 1:100, Proteintech) and SMA (ab7817, 1:100, Abcam) overnight at 4 °C. Then, sections were incubated with secondary antibodies. Images were captured by a fluorescence microscope (Zeiss) and analyzed by ImageJ (NIH).

### 2.9. Western Blot

By putting the samples into RIPA lysis buffer containing a protease inhibitor cocktail (Roche, Shanghai, China), the proteins were extracted, and a BCA protein assay (Beyotime, Nanjing, China) was used to quantify their concentration. Equal amounts of protein were subjected for electrophoresis.

First, proteins were transferred onto polyvinylidene difluoride membranes (Millipore, Billerica, MA, USA), which were blocked in 5% nonfat milk and distilled water for 2 h at room temperature. Membranes were incubated overnight with diluted primary antibody at 4 °C. Primary antibodies were MMP2 (10373‐2‐AP, 1:500, Proteintech), MMP9 (MMP9, 10375‐2‐AP, 1:500, Proteintech), collagen 1 (14695‐1‐AP, 1:1000, Proteintech), Collagen 3 (68320‐1‐lg, 1:5000, Proteintech), IL‐1*β* (16806‐1‐AP, 1:2000, Proteintech), TGF‐*β*2 (sc‐374658, 1:100, Santa Cruz), TNF‐*α* (17590‐1‐AP, 1:1000, Proteintech), CD 31 (ab222783, 1:1000, Abcam), fibronectin (ab2413, 1:1000, Abcam), vimentin (10366‐1‐AP, 1:10000, Proteintech), VE‐cadherin (27956‐1‐AP, 1:1000, Proteintech), SMA (ab7817, 1:1000, Abcam), Smad2 (12570‐1‐AP, 1:2000, Proteintech), p‐Smad2 (ab300079, 1:1000, Abcam), Smad3 (66599‐1‐lg, 1:1000, Proteintech), p‐Smad3 (ab63403, 1:500, Abcam), and GAPDH (10494‐1‐AP, 1:5000, Proteintech). After washing membranes with secondary antibody (Proteintech), bands were visualized with ChemiDoc XRS (Bio‐Rad). Band densities were evaluated in ImageJ (NIH).

### 2.10. Statistical Analysis

Statistical analyses were performed in GraphPad Prism 8.0. All results are expressed as the mean ± standard deviation (SD) and were analyzed either with one‐way ANOVA or independent Student’s *t*‐test. Significance was set at *p*  < 0.05.

## 3. Results

### 3.1. Hemodynamic Data, Body Weights, and Organo‐Somatic Indices (OSI) of the Penis in all Rats

As shown in Table 1, there were no significant differences of body weights and OSI of the penises among groups, and it was found that L‐NAME increased SBP, DBP, and MAP in rats compared to the control group, while intracavernosal injection barely had an effect on the development of systemic hypertension. Hypertension (defined as SBP > 140 mmHg) was successfully induced in 100% of L‐NAME–treated rats (24/24). All hypertensive rats exhibited significant ED, evidenced by reduced ICPmax/MAP (Figure 1E) and histological alterations (Figures 1 and 2).

Figure 1tSVF therapy improved erectile function and restored endothelial content in hypertensive rat models. (A–D) ICP responses to electrostimulation in the NC, PBS, cSVF, and tSVF groups. (E) Maximum ICP to MAP ratio responses to electrostimulation in the four groups. (F) Total ICP to MAP ratio responses to electrostimulation in the four groups. (G, I) Immunofluorescence staining of vWF in the penile of NC, PBS, cSVF, and tSVF groups. (H, J) Immunofluorescence staining of eNOS in the penile of four groups. Data are shown as mean ± SD. “ ^∗^”, “ ^∗∗^”, and “ ^∗∗∗^” are represented as statistically different as *p*  < 0.05, *p*  < 0.01, and *p*  < 0.001, respectively. Those with horizontal lines represent statistical differences from the ends of the horizontal lines, and those without horizontal lines represent statistical differences compared to the PBS. Scale bar = 100 μm.

**Figure:**
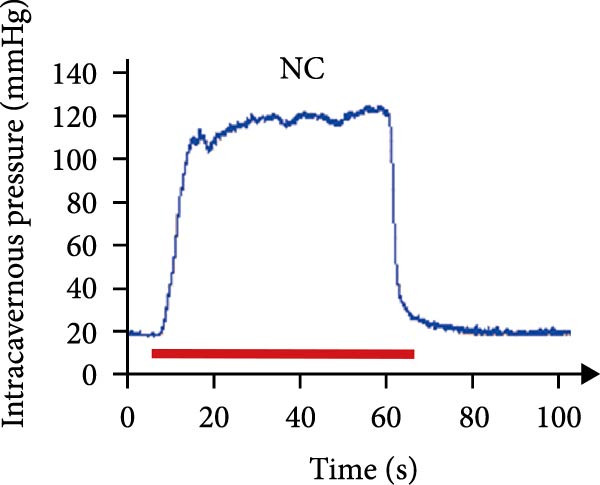
(A)

**Figure:**
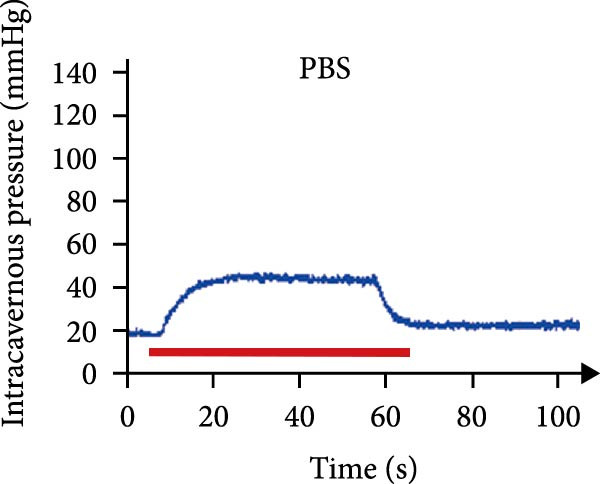
(B)

**Figure:**
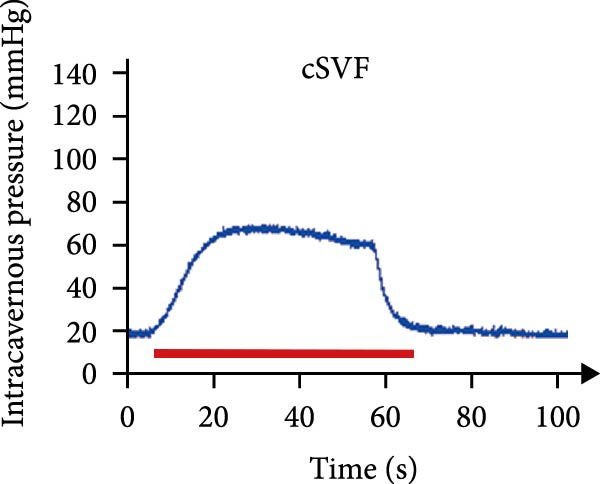
(C)

**Figure:**
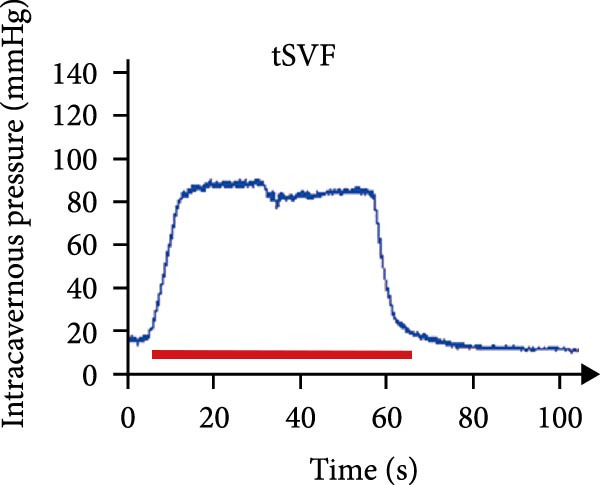
(D)

**Figure:**
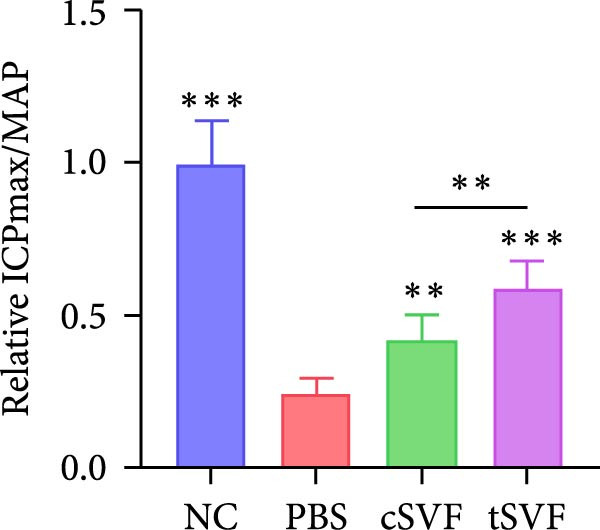
(E)

**Figure:**
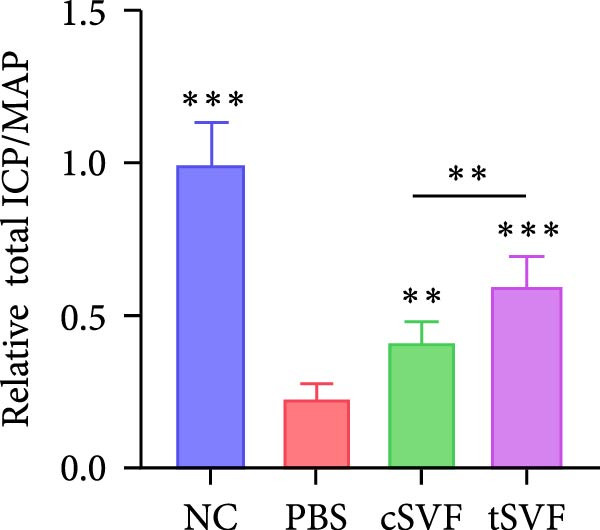
(F)

**Figure:**
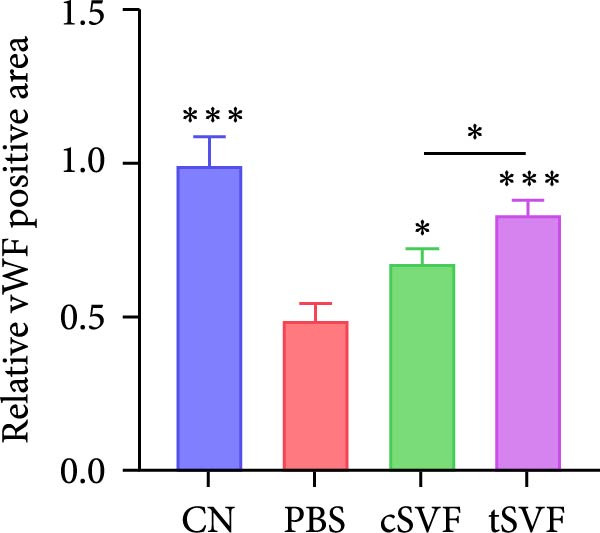
(G)

**Figure:**
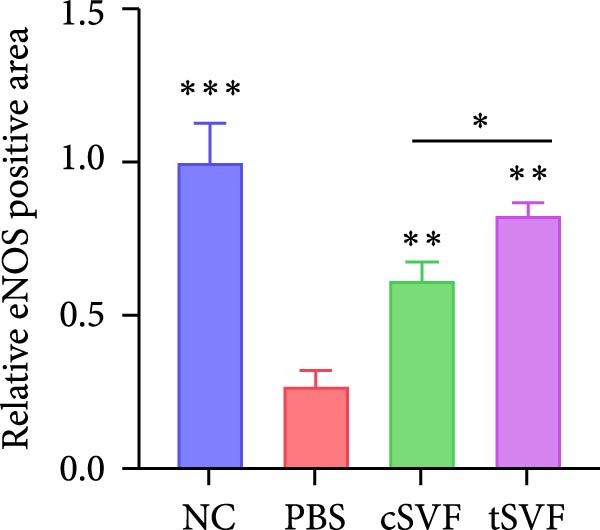
(H)

**Figure:**
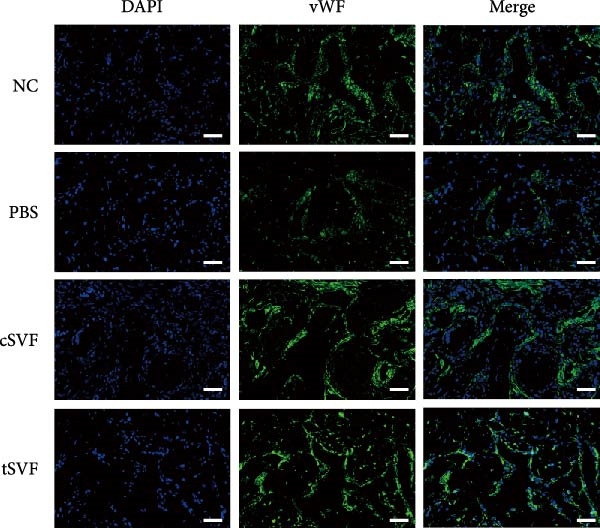
(I)

**Figure:**
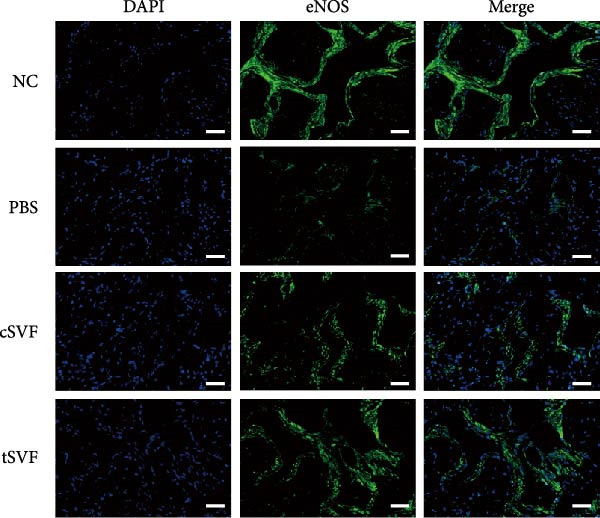
(J)

Figure 2tSVF ameliorated the collagen deposition in the penis of hypertensive rats. (A, C) Masson’s trichrome staining of the penile used to assess the smooth muscle/collagen ratio in the penile of NC, PBS, cSVF, and tSVF groups. (B, D) Immunohistochemical staining for MMP2 and MMP9 expression of penile sections from four groups. (E) Protein expression of collagen 3, collagen 1, MMP2, and MMP9 from four groups. Data are shown as mean ± SD. “ ^∗^”, “ ^∗∗^”, and “ ^∗∗∗^” are represented as statistically different as *p*  < 0.05, *p*  < 0.01, and *p*  < 0.001, respectively. Those with horizontal lines represent statistical differences from the ends of the horizontal lines, and those without horizontal lines represent statistical differences compared to the PBS. Scale bar = 100 μm.

**Figure:**
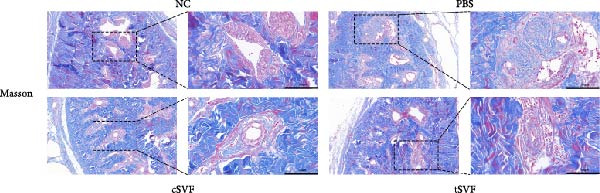
(A)

**Figure:**
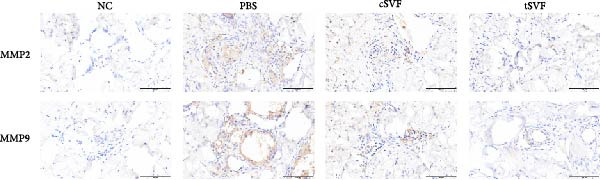
(B)

**Figure:**
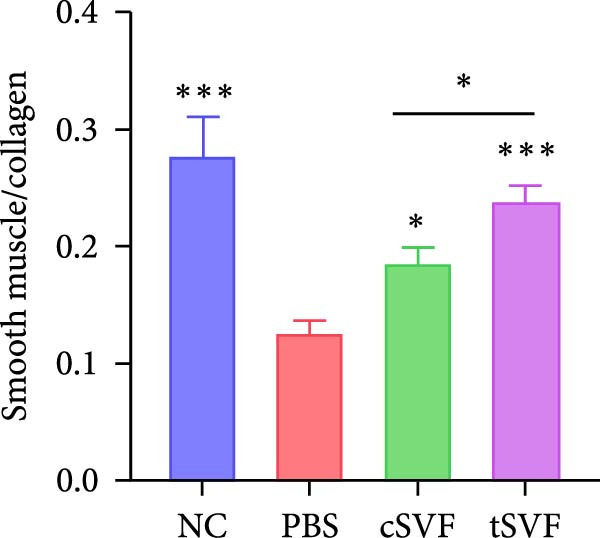
(C)

**Figure:**
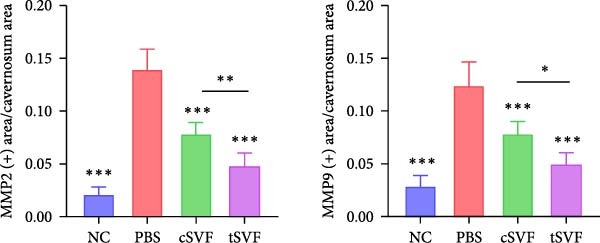
(D)

**Figure:**
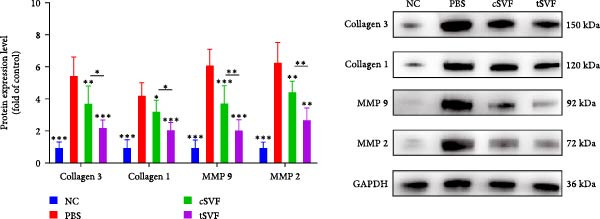
(E)

**Table 1:** Hemodynamic data, body weights, and organo‐somatic indices (OSI) of the penis in all rats.

Parameters	NC	PBS	cSVF	tSVF
SBP (mmHg)	110.0 ± 12.06	164.9 ± 16.49^c^	160.7 ± 20.13^c^	155.8 ± 19.18^c^
DBP (mmHg)	87.6 ± 11.17	123.1 ± 4.87^b^	117.3 ± 23.09^a^	116.6 ± 14.66^a^
MAP (mmHg)	95.1 ± 11.45	137.1 ± 7.68^c^	131.8 ± 21.02^b^	129.9 ± 15.37^b^
Body weight (g)	469.6 ± 15.38	458.5 ± 18.16	467.6 ± 12.77	466.5 ± 18.70
OSI of the penis (g)	0.10 ± 0.020	0.09 ± 0.014	0.09 ± 0.015	0.10 ± 0.016

*Note*: Data are expressed as mean ± SD, (*n* = 8/group).

Abbreviations: DBP, diastolic blood pressure; MAP, mean arterial pressure; OSI, organo‐somatic indices; SBP, systolic blood pressure.

^a^ *p*  < 0.01 significant difference with NC.

^b^ *p*  < 0.001 significant difference with NC.

^c^ *p*  < 0.0001 significant difference with NC.

### 3.2. Extraction and Characterization of tSVF

The extraction process of tSVF is shown in Figure 3A. The volume of tSVF was <15% of the original fat volume, and it can be injected into cavernosa through an insulin needle (BD U‐40 insulin syringe) (Figure 3A[f]). After being digested by collagenase, the flow cytometry suggested that tSVF comprised mainly heterogeneous cell populations expressing hematopoietic (CD11b/c and CD45), mesenchymal (CD29, CD90, and CD106), and endothelial (CD31 and VEGFR2) markers (Figure 3B). Compared to cSVF, the total live cell number of all the subpopulations decreased during the processing. Among them, the number of hematogenous cells decreased most sharply. Approximately 58.1% of the ADSCs and 49.2% of the endothelial cells remained in the final product (Figure 3C). After 2‐week induction by L‐NAME, we injected PBS, cSVF, and tSVF into the cavernosa of hypertensive rat models, respectively, and therapeutical effects were evaluated after 4 weeks (Figure 3D).

Figure 3(A) Extraction and injection of tSVF. (a) Sheared fat particles; (b) fat passed through tSVF converter; (c, d) emulsified celiac mixture before and after centrifuging; (e) extraction of the middle layer of centrifuged emulsified celiac mixture; (f) injection of tSVF with a BD U‐40 insulin syringe. (B) Representative flow cytometry histograms of tSVF digested by collagenase. (C) The cell numbers isolated from different processing conditions. (D) Schematic diagram of the process of extraction of cSVF and tSVF and experimental protocol for the treatment of hypertensive ED.

**Figure:**
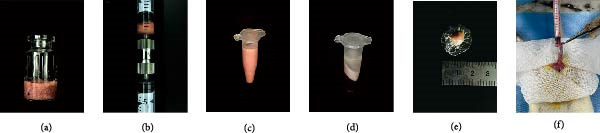
(A)

**Figure:**
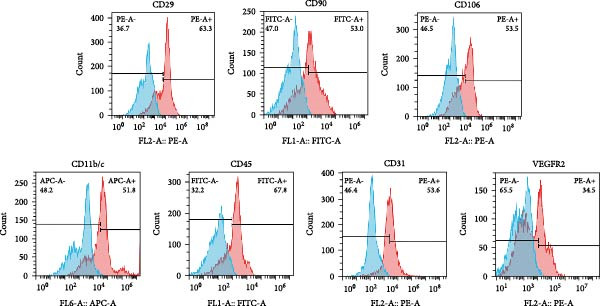
(B)

**Figure:**
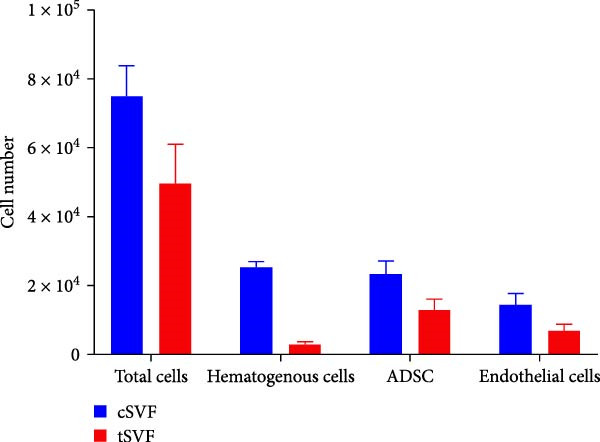
(C)

**Figure:**
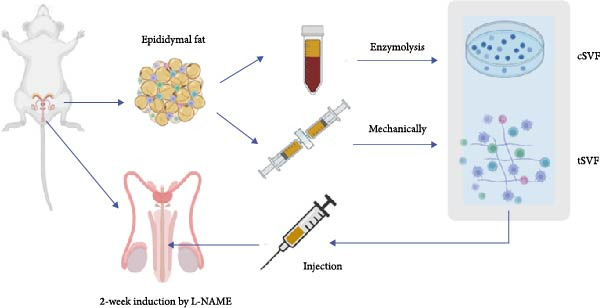
(D)

### 3.3. tSVF Therapy Improved Erectile Function and Restored Endothelial Content in Hypertensive Rat Models

We performed erectile function assays at 4 weeks after establishing the PBS, cSVF, tSVF, and control groups among L‐NAME rats (Figure 1A–D). Compared with normal rats, the PBS group had higher MAP but significantly lower ICP; therefore, ICPmax/MAP and total ICP/MAP decreased considerably from control levels in the PBS group (Figure 1E–F). Additionally, the cSVF and tSVF groups had significantly higher ICP at erection than did the PBS group, which means SVF treatment can reverse the ED induced by hypertension. When comparing ICPmax/MAP and total ICP/MAP of two SVF‐treated groups, the tSVF group was higher than the cSVF, indicating that the tSVF group received more effective treatment. Moreover, immunofluorescence showed that hypertension significantly lowered the expression of vascular endothelial cell marker vWF, a trend reversed under both cSVF and tSVF treatments (Figure 1G, I). The detection of another important endothelial marker, eNOS, showed consistent results (Figure 1H,J). Although the two are both effective in restoring endothelial content in the penis of hypertensive rats, there were differences between the cSVF group and tSVF group. The tSVF group was closer to control levels, whether it is the expression of vWF or eNOS.

### 3.4. tSVF Ameliorated the Collagen Deposition in the Penis of Hypertensive Rats

Normal penile erection requires intact, functioning smooth muscles and vascular endothelial tissues. The results of Masson’s trichrome staining revealed a significantly lower smooth muscle‐to‐collagen ratio in the PBS group than in the control group (Figure 2A,C). Conversely, immunohistochemistry measurements of fibrosis indicators MMP2 and MMP9 in the PBS group showed significantly more expression compared to the control level due to the stimulation of hypertension (Figure 2B,D). However, after treating with cSVF or tSVF, the smooth muscle‐to‐collagen ratio was reversed, with decreased expression of MMP2 and MMP9, indicating the recovery of smooth muscles in penile tissues. In addition, we used western blot to detect the expression of collagen 1, collagen 3, MMP2, and MMP9 in penile tissue of rats in each group, and the results showed that the above proteins suggesting fibrosis were all elevated in the PBS group and were all reducible after cSVF and tSVF treatment (Figure 2E).

### 3.5. tSVF Therapy Inhibited EndMT in Penis of Hypertensive Rats

During EndMT, endothelial cells lose their property factors and differentiate into mesenchymal cells. To determine the presence of transitional EndMT cells in the penile cavernous of hypertensive rats, the distribution and expression of mesenchymal cell marker, SMA (green fluorescent signals), and endothelial cell marker, vWF (red fluorescent signals), were analyzed by indirect immunofluorescence doublestaining assay. As seen in Figure 4A, double‐positive staining for vWF and SMA (pointed by white arrow) was distributed in the partial endothelium of remodeled cavernous sinus, and the colocalization was reduced upon the treatment of cSVF and tSVF. Moreover, we observed significant upregulation of fibronectin and vimentin as downregulation of VE‐cadherin and CD31 at the protein level (Figure 4B,C). Treatment with cSVF and tSVF significantly reversed this phenotype. Considering the occurrence of EndMT has a strong relationship with TNF‐*α*, TGF‐*β*2, and IL‐1*β*, immunofluorescence staining and western blotting of TNF‐*α*, TGF‐*β*2, and IL‐1*β* in the cavernosa showed that their expression was much more than in the PBS group than in the control, and both cSVF and tSVF could reduce the production of inflammatory factors (Figure 5). The results indicated that SVF had a therapeutic effect on inhibiting the inflammation related with the occurrence of EndMT. Moreover, after comparing all EndMT indicators in the two groups, tSVF performed better than cSVF. Consistent with the former results, the intensity of TNF‐*α*, TGF‐*β*2, and IL‐1*β* was weaker in the tSVF group than the cSVF group, which means tSVF had a stronger inhibitory effect than cSVF.

Figure 4tSVF therapy inhibited EndMT in hypertensive rats. (A) Confocal laser microscopy analysis shows representative images of vWF (red) and SMA (green) double staining in penis sections from NC, PBS, cSVF, and tSVF groups. Double‐positive staining for vWF and SMA is pointed to by white arrows. (B, C) Fibronectin, VE‐cadherin, CD31, and vimentin protein expression in penile tissue was determined by western blot in the four groups. Data are shown as mean ± SD. “ ^∗^”, “ ^∗∗^”, and “ ^∗∗∗^” are represented as statistically different as *p*  < 0.05, *p*  < 0.01, and *p*  < 0.001, respectively. Those with horizontal lines represent statistical differences from the ends of the horizontal lines, and those without horizontal lines represent statistical differences compared to the PBS. Scale bar = 100 μm.

**Figure:**
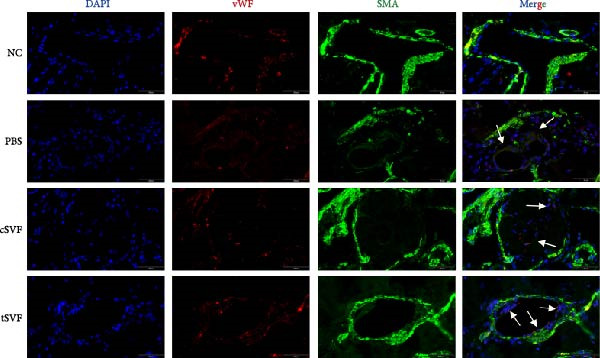
(A)

**Figure:**
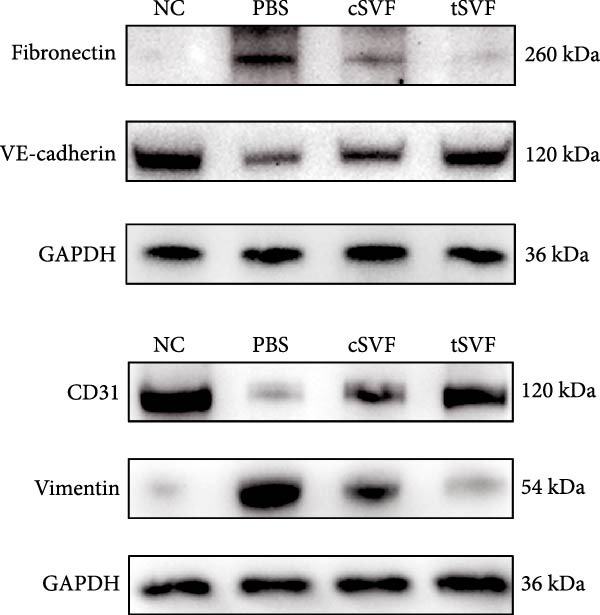
(B)

**Figure:**
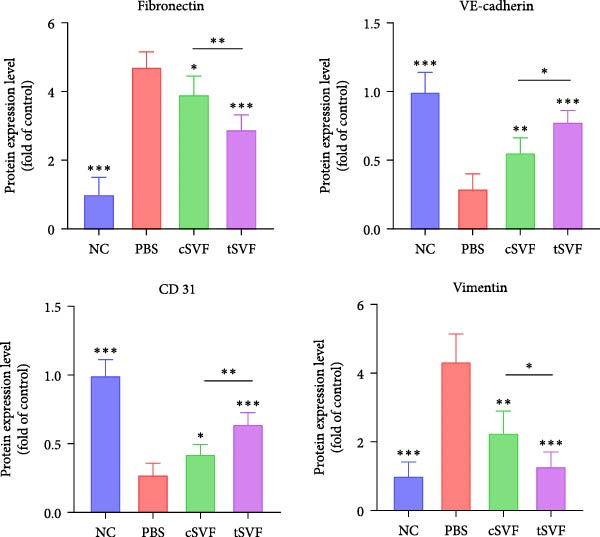
(C)

Figure 5Transplantation of tSVF inhibits activation of EndMT‐related inflammation in hypertensive rats. (A–D) Immunofluorescence staining of TNF‐*α*, TGF‐*β*2, and IL‐1*β* in the penile of NC, PBS, cSVF, and tSVF groups. (E, F) TNF‐*α*, TGF‐*β*2, and IL‐1*β* protein expression in penile tissue was determined by western blot in the four groups. Data are shown as mean ± SD. “ ^∗^” and “ ^∗∗^” are represented as statistically different as *p*  < 0.05 and *p*  < 0.01, respectively. Those with horizontal lines represent statistical differences from the ends of the horizontal lines, and those without horizontal lines represent statistical differences compared to the PBS. Scale bar = 100 μm.

**Figure:**
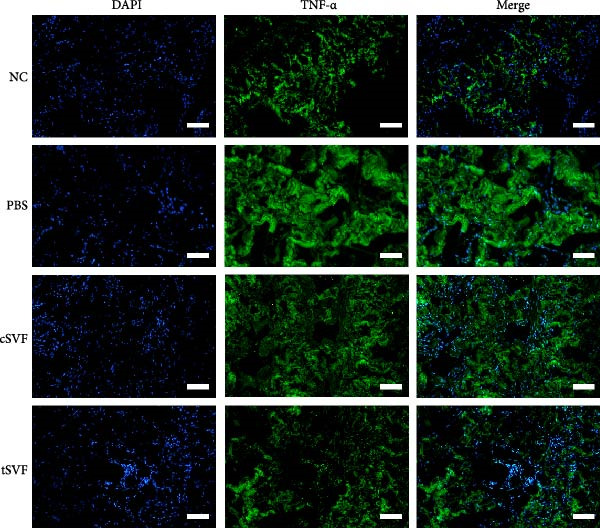
(A)

**Figure:**
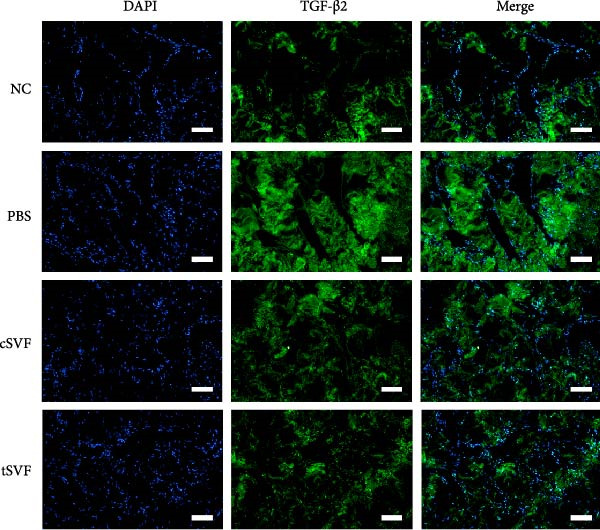
(B)

**Figure:**
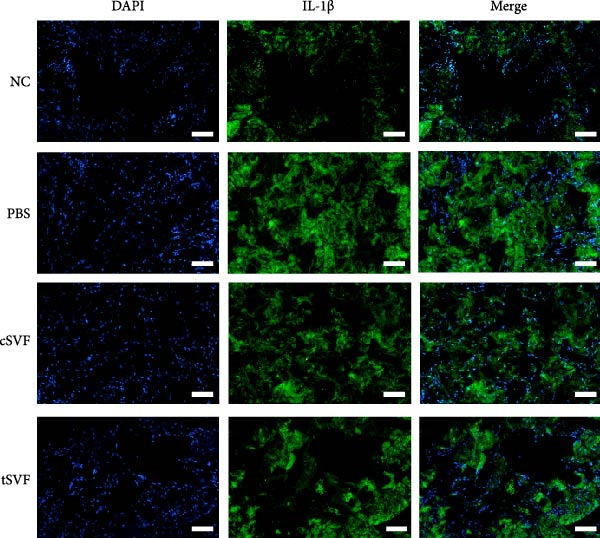
(C)

**Figure:**
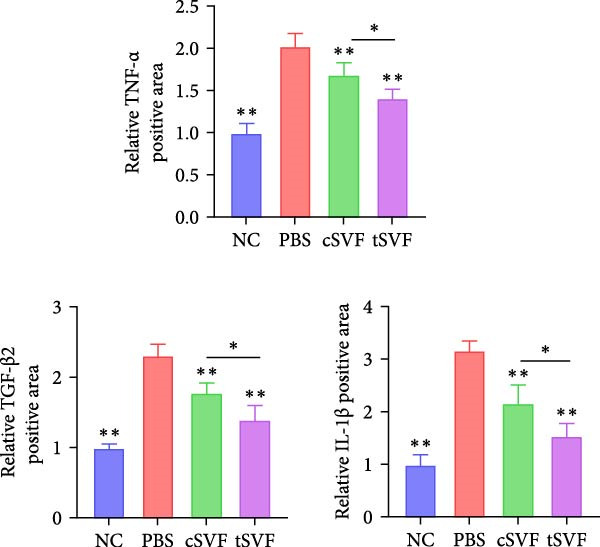
(D)

**Figure:**
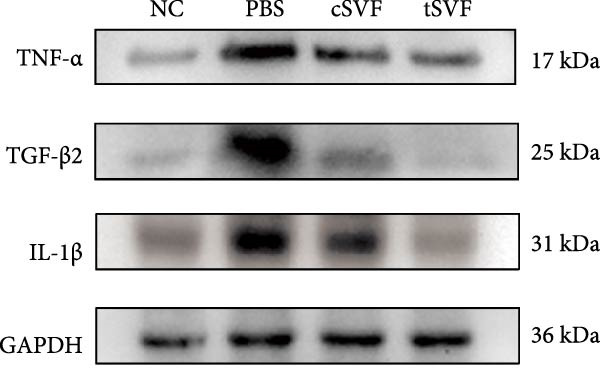
(E)

**Figure:**
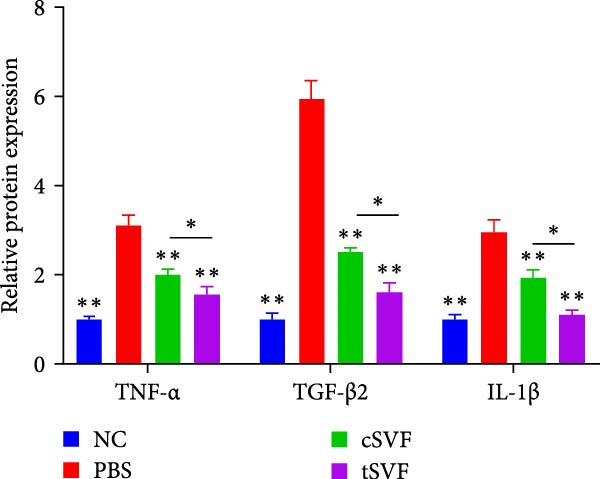
(F)

### 3.6. tSVF Suppresses EndMT in L‐NAME‐Induced HUVECs via Inhibiting the TGF‐*β*2–Smad2/Smad3 Pathway

To further validate and explore the conclusion that tSVF improves endothelial function by inhibiting EndMT and its underlying mechanisms, we simulated the pathological process of endothelial cell damage in the penile cavernosum of hypertensive rats using L‐NAME–induced endothelial dysfunction in HUVECs, following the protocol described in the Methods section. During induction, cells were cocultured with cSVF or tSVF and compared to normal control (NC) and untreated groups. Results demonstrated that both cSVF and tSVF treatments significantly attenuated the fibrotic morphological changes in HUVECs. Treated cells also exhibited reduced expression of fibrosis‐characteristic proteins (SMA) while preserving endothelial markers (VE‐cadherin) compared to the untreated group (Figure 6A,B). Quantitative analysis of endothelial‐associated proteins (CD31 and VE‐cadherin) and fibrosis‐associated proteins (SMA and vimentin) further corroborated these findings (Figure 6C,D), showing trends consistent with EndMT–related protein expression in cavernous endothelial cells of hypertensive rats. Previous experiments confirmed differential TGF‐*β*2 expression in rat cavernous tissues, where its upregulation promoted EndMT. To elucidate TGF‐*β*2’s mechanistic role in EndMT, we examined downstream signaling molecules. Results revealed that treated cells (cSVF/tSVF) showed significantly reduced p‐Smad2/p‐Smad3 protein expression compared to the untreated group (Figure 6E,F). Critically, both immunofluorescence and protein quantification of EndMT markers, as well as measurements of TGF‐*β*2 and its downstream effectors, indicated that tSVF treatment yielded results closer to the NC group than cSVF treatment. This aligns with *in vivo* experimental outcomes.

Figure 6tSVF suppresses EndMT in L‐NAME–induced HUVECs via inhibiting the TGF‐*β*2–Smad2/Smad3 pathway. (A–C) Immunofluorescence costaining for VE‐cadherin (red) and SMA (green) revealed differences in cellular morphology and the extent of EndMT in HUVECs across the NC, L‐NAME, cSVF, and tSVF groups. (C–E) CD31, VE‐cadherin, SMA, vimentin, TGF‐*β*2, Smad2, p‐Smad2, Smad3, and p‐Samd3 protein expression in HUVECs was determined by western blot in the four groups. Data are shown as mean ± SD. “ ^∗^”, “ ^∗∗^”, and “ ^∗∗∗^” are represented as statistically different as *p*  < 0.05, *p*  < 0.01, and *p*  < 0.001, respectively. Those with horizontal lines represent statistical differences from the ends of the horizontal lines, and those without horizontal lines represent statistical differences compared to the L‐NAME.

**Figure:**
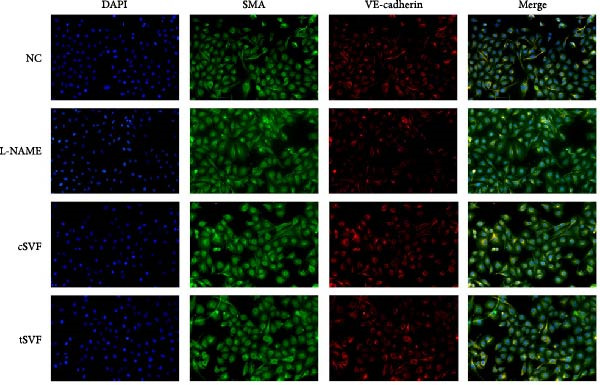
(A)

**Figure:**
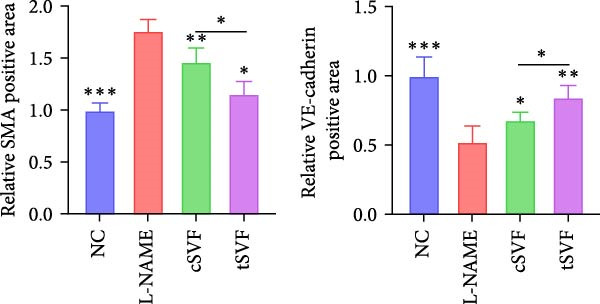
(B)

**Figure:**
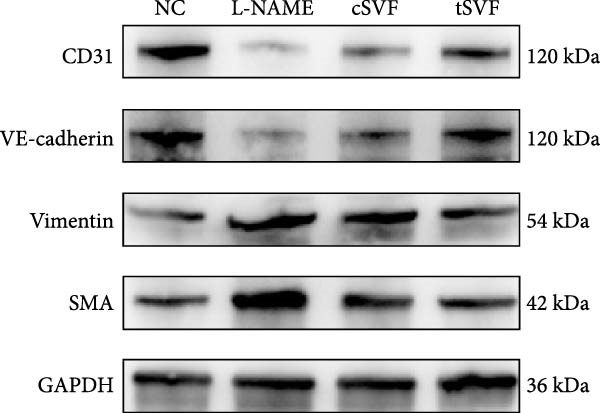
(C)

**Figure:**
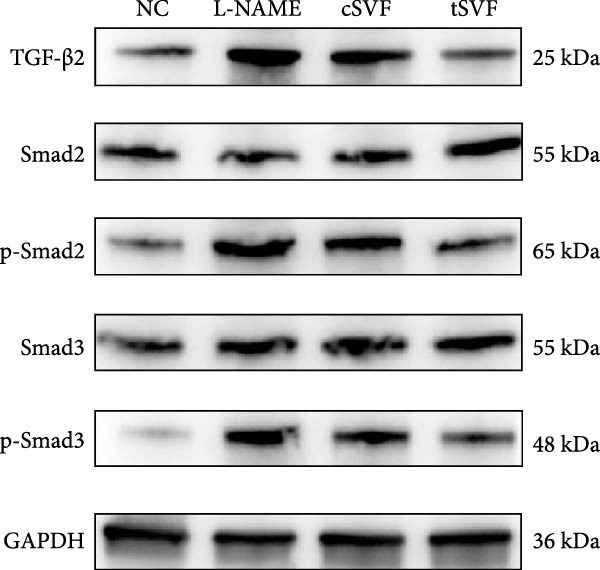
(D)

**Figure:**
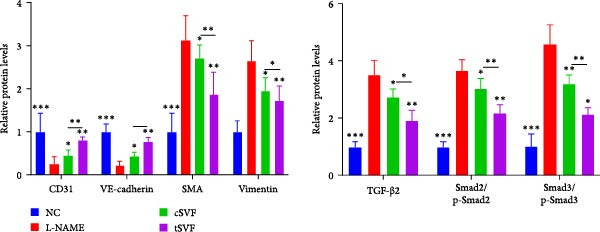
(E)

### 3.7. tSVF Manifested the Property of Longevity and Transdifferentiation in the Penis

To determine whether cSVF and tSVF survived, proliferated, and transdifferentiated in vivo, immunofluorescence staining was used, and the previously labeled cSVF and tSVF could be observed as red under fluorescence microscopy. At 1, 2, and 4 weeks after the injection of cSVF and tSVF, cavernous tissue of the two groups was costained with PCNA, vWF, and eNOS, respectively. As shown in Figure 7D, although both tSVF and cSVF exhibited a general declining trend in retention rates over time, the quantitative analysis of CM‐Dil–positive areas within the corporal cavernosa at each matched time point revealed significantly larger preserved areas in the tSVF group compared to the cSVF group. These results suggest that tSVF was maintained more completely and persisted longer within the tissue, thereby enhancing its regenerative capacity and therapeutic efficacy in repairing damaged tissue. Furthermore, the costaining with PCNA, a proliferation indicator, revealed that PCNA–positive cells were mainly present around cSVF and tSVF but not in themselves after 1 week of injection. By 2 and 4 weeks, tSVF was expressing PCNA itself accompanied by the disappearance of cSVF (Figure 7A), suggesting that the tSVF not only survived but also maintained a proliferative state in the cavernous body as well. In addition, immunofluorescence staining demonstrated that the tSVF costained with eNOS from 2 weeks after injection and expressed vWF at 4 weeks (Figure 7B,C), suggesting that tSVF may differentiate into endothelial cells. However, it is rare to discover the coexpress area of CM‐Dil–labeled cSVF with these indicators. In summary, tSVF possessed the capability of longevity and transdifferentiation in the penis, which is exactly what cSVF lacks.

Figure 7The tSVF showed longevity and transdifferentiation potential in the hypertensive rat ED model. (A–C) Some Dil‐labeled cells expressing PCNA (A), vWF (B), or eNOS (C) were detected in tSVF group on weeks 1, 2, and 4 after intracavernous injection. The cSVF was disappeared at the 4th week and showed nearly no longevity and transdifferentiation potential in the hypertensive rat ED model. (D) Despite a general decline in cell retention rates over time in both groups, the tSVF group maintained a higher retention rate than the cSVF group at each corresponding time point. Data are shown as mean ± SD. “ ^∗^” and “ ^∗∗^” are represented as statistically different as *p* < 0.05 and *p* < 0.01, respectively. The boxed region is shown at 50x magnification. Scale bar = 250 μm.

**Figure:**
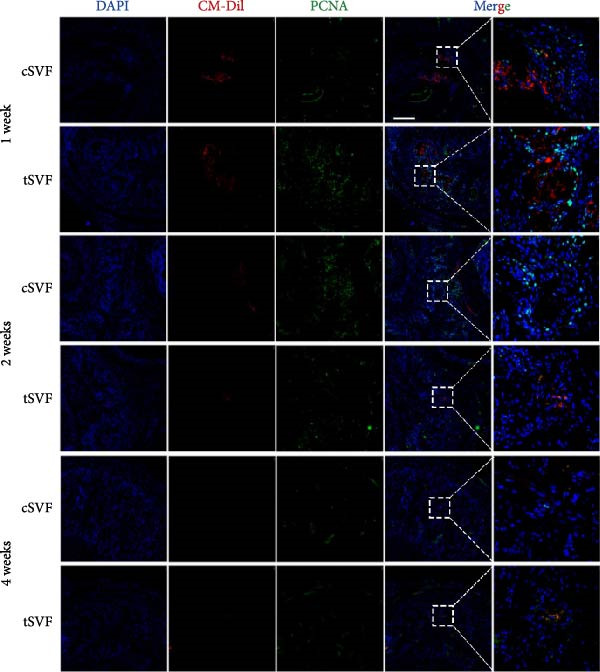
(A)

**Figure:**
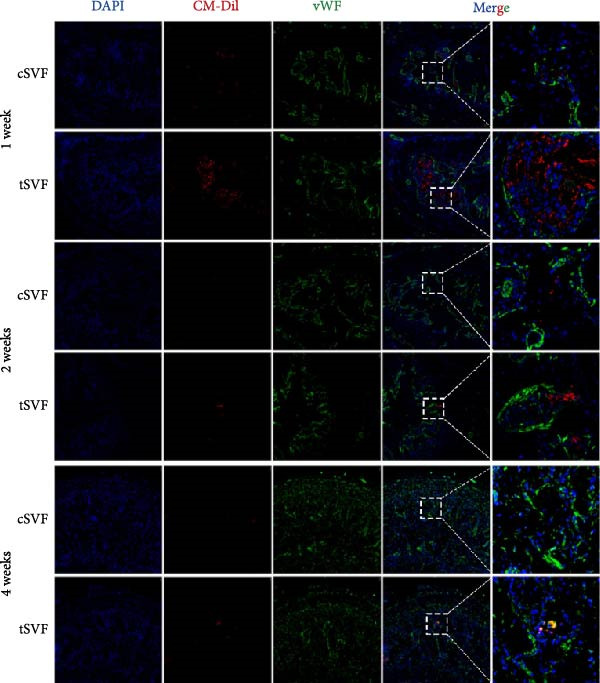
(B)

**Figure:**
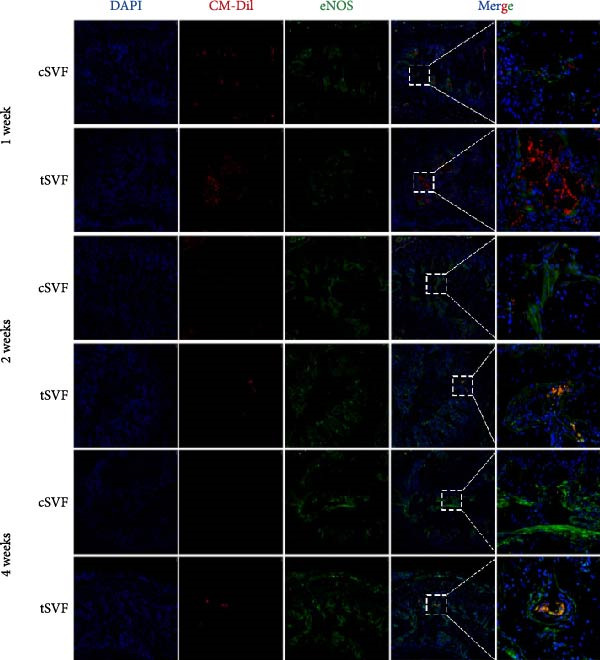
(C)

**Figure:**
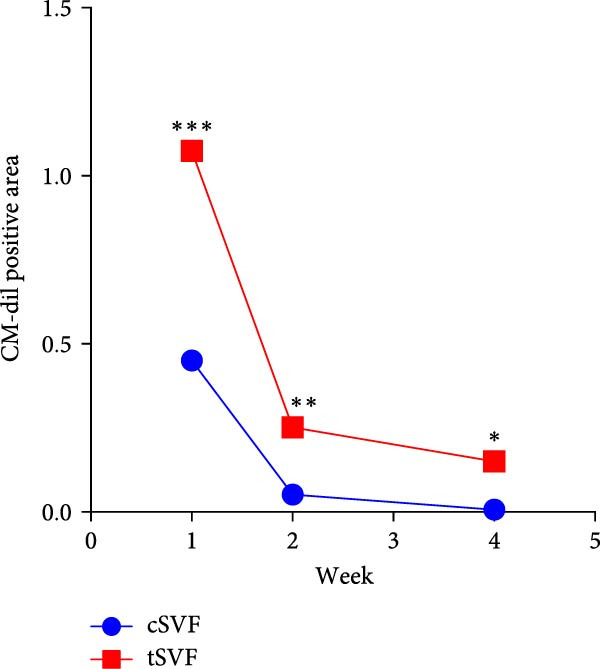
(D)

## 4. Discussion

The vascular endothelium maintains homeostasis via vasoactive relaxing factors (nitric oxide, prostacyclin, and endothelium‐derived hyperpolarizing factor) and contracting factors (superoxide anion, endothelin‐1, and constrictive prostaglandins) [31]. Under hypertension, contracting factors increase to induce an inflammatory response and oxidative stress in vascular endothelial cells, manifesting as vascular structural remodeling and tissue fibrosis [32, 33]. When hypertension affects penile endothelium, smooth muscles of the corpus cavernosa cannot fully dilate during erection, restricting blood flow and impairing erectile function [34, 35]. In this study, we used L‐NAME to explore the effects of tSVF on hypertension‐induced ED. We chose L‐NAME based on reports that chronic oral treatment of male rats with a NOS inhibitor is a relevant peripheral ED model for evaluating potential ED drug effects [36–38]. As hypothesized, L‐NAME successfully induced a BP increase in rats. Follow‐up experiments also demonstrated that L‐NAME–treated rats had significantly impaired erectile function, with the cavernous tissue exhibiting fibrosis and inflammation. Our sample size (*n* = 8/group) is consistent with comparable rodent studies [39–41], and the L‐NAME model reliably induced hypertension and ED, as demonstrated by functional and molecular pathology. Future studies may expand cohorts to validate secondary mechanisms.

Previous studies have proved that the stem cell treatment can exert therapeutic effects in animal models of diabetes and neurological ED by improving the vascular function of cavernous endothelium and suppressing cavernous fibrosis [42–44]. The direct differentiation of stem cells into cavernous endothelial cells or smooth muscle cells is rarely observed; thus, it is generally believed that the therapeutic role of stem cells primarily relies on their paracrine effects [44, 45], by which stem cell exerts its therapeutic effect to reduce the apoptotic and inflammatory response levels related to ED risk factors [46, 47]. The therapeutic effect of tSVF has been validated in multiple disease models, including osteoarthritis [48, 49], anal sphincter incontinence [50], and dermal injury [51]. However, there are no studies that have explored the effects of tSVF on ED to our knowledge. So we established hypertensive rat models, and our study has now confirmed the positive effect of tSVF by measuring the ICPmax/MAP and total ICP/MAP. We then further evaluated the cavernous vascularization and fibrosis condition in each group, and the results showed that tSVF could reduce fibrosis and restore endothelial content in the penis after hypertension.

A meta‐analysis of research from 1991 to 2022 concluded that vascular endothelial cells are a key factor in ED pathogenesis and also that stem cell therapy has considerable potential, given the known effects on improving endothelial function [52]. Among cardiovascular diseases, EndMT is considered an important pathological mechanism that can cause endothelial cell dysfunction [53]. The transition increases blood vessel permeability during the early stages of inflammation and promotes inflammation‐mediated angiogenesis. A persistently pathological environment can cause overactive EndMT that stiffens tissues and hampers normal function [54]. During EndMT, endothelial cell‐specific markers are gradually replaced by mesenchymal markers that migrate, invade, and proliferate [55]. In our study, we found that the expression of vascular endothelial markers decreased in the penis of hypertensive rats, accompanied by the lower smooth muscle‐to‐collagen ratio, so we hypothesized that EndMT occurred in the cavernosa of the penis in hypertensive rats. To verify this conjecture, we detected the expression of the marker of EndMT with western blotting. The result showed that the expression of VE‐cadherin and CD31 decreased in the penis of hypertensive rats, which is a component of endothelial cell‐to‐cell adhering junctions, while the protein amount of the fibroblast markers increased. To specifically identify and quantify EndMT within endothelial cells in situ, we used combinations of markers to precisely track the fate of endothelial cells. vWF (endothelial markers) + SMA (mesenchymal markers): The presence of double‐positive cells is a gold‐standard criterion for identifying cells undergoing EndMT. Our figures (Figure 4A) clearly show an increased population of these double‐positive cells in the corpora cavernosa of hypertensive rats compared to NC. Critically, treatment with tSVF significantly reduced the prevalence of these transitional cells, providing direct visual evidence that tSVF inhibits EndMT specifically within the endothelial lineage. Additionally, we used HUVECs to simulate the pathological changes of corpus cavernosus endothelial cells induced by L‐NAME and evaluated the morphological characteristics of endothelial cells and the expression levels of EndMT markers in each group using immunofluorescence staining. We then performed Western blot analysis on lysates from these groups. This approach allows for a direct assessment of protein expression changes exclusively in endothelial cells. The results (Figure 6) corroborate our whole‐tissue findings. They confirm that the shifts in EndMT marker expression observed in the whole tissue partly originate from the endothelial cell population, strengthening our conclusion that EndMT is a significant pathological process in hypertensive ED and a key target of tSVF therapy.

We should clarify that our data demonstrate EndMT is a contributing mechanism; the overall fibrotic phenotype likely results from a combination of EndMT‐derived myofibroblasts, activation and proliferation of resident fibroblasts, or phenotypic modulation of vascular smooth muscle cells. Considering previous literature has reported that endothelial cells constitute only ~6.93% ± 0.86% of the rat corpus cavernosum [56], our claim is not that EndMT is the sole source of fibrosis, but that it is a significant and previously underinvestigated pathway in ED, and that its inhibition by tSVF constitutes a key part of its therapeutic mechanism.

In order to explore the mechanism underlying the EndMT occurrence in the penis of hypertensive rats, we detected the expression level of inflammatory factors associated with EndMT. Inflammatory factors have been proved to induce vascular remodeling, inflammatory response, fibrosis, and loss of endothelial function, leading to structural and functional abnormalities in multiorgan fibrosis such as heart, lung, and kidney [57–60]. Although the underlying mechanisms are still far from being totally understood, TGF‐*β* is currently the main and most well‐studied EndMT inducer [61–63]. TGF‐*β* has three highly conserved isoforms which are known as TGF‐*β*1, TGF‐*β*2, and TGF‐*β*3. After binding to the TGF‐*β* receptor types Ⅰ and ⅠⅠ, the receptors are activated, and the signals are transduced into the cell [64]. The three isoforms associated with EndMT through different actions. TGF‐*β*1 has been reported to be involved in EndMT [65] during which the transcription factors Snail and Slug are overexpressed, leading to the suppression of cell‐to‐cell adhesions. Moreover, there is evidence that microRNAs may participate in the regulation of EndMT, such as miR126 [66]. It has been reported that EndMT can be induced by TGF‐*β*2 via the activation of the Smad protein cascade, extracellular signal‐regulated kinases 1/2 (ERK 1/2), PI3K, and p38 mitogen‐activated protein kinase (MAPK) pathways [67]. In our study, we measured the expression levels of TGF‐*β*2, a key initiator of EndMT, in endothelial cells across different experimental groups, and further assessed the phosphorylation levels of its downstream signaling molecules, Smad2/3. Based on these findings, we propose that the activation of this signaling pathway represents an important molecular mechanism underlying EndMT in cavernous tissue. Besides, Ghosh et al. [68] found that the expression of miRNAs was altered during TFG‐*β*2–induced EndMT. They found that miR125b, let‐7c, let‐7g, miR‐21, miR‐30b, and miR‐195 were upregulated, while miR‐122a, miR‐127, miR‐196, and miR‐375 were downregulated. However, the role of these miRNAs on EndMT needs to be further explored. TGF‐*β*3 is more discussed during the embryonic stage of development but not described very often under the context of endothelial transition [64]. Sabbineni et al. [69] compare the dose‐dependent effects of TGF*β*1, TGF*β*2, and TGF*β*3 on EndMT and found that the effect of TGF*β*2 was the most obvious. Moreover, after treatment with 1 ng/mL TGF*β*1 and TGF*β*3, but not TGF*β*2, they found that the expression of TGF*β*2 increased, indicating that EndMT with TGF*β*1 and TGF*β*3 treatments was due to the secondary effects through TGF*β*2 secretion. Therefore, they concluded that TGF*β*2 is the most potent inducer of EndMT and that TGF*β*1– and TGF*β*3–induced EndMT necessitates a paracrine loop involving TGF*β*2. IL‐1*β* is a cytokine mainly produced by activated macrophages. It also involved in the process of endothelial fibrosis. The conclusion that IL‐1*β* can produce phenotypic changes in EC was first proposed in 1997 [70], and the results were further confirmed by Chaudhuri et al. [71]. Apart from inducing EndMT itself, Maleszewska et al. [72] demonstrated that IL‐1*β* can induce EndMT in HUVECs synergistically in the presence of TGF‐*β* in an NF*κ*B–dependent manner. Additionally, after stimulation with IL‐1*β* and TGF‐*β* simultaneously, HUAECs induced a decrease in the endothelial NOS (eNOS) and vWF [72], which is consistent with the results of our experiments. TNF‐*α* has been found that TNF‐*α* is associated with skin [71] and intestinal fibrosis [73]. In ECs, TNF‐*α* can induce the expression of adhesion proteins as a consequence of the inflammatory stimulus, thus activating EndMT [74]. We found that tSVF successfully depressed the expression level of TGF‐*β*2, TNF‐*α*, and IL‐1*β*, which may explain the inhibition of EndMT in corpora cavernosa of hypertensive rats. However, the potential mechanisms underlying how tSVF acts under the stimulation of these inflammatory cytokines need further investigation.

Our study also compared cSVF with tSVF for the treatment of hypertensive ED. Notably, ICPmax/MAP and total ICP/MAP suggested that the benefits were greater in the tSVF group than in the cSVF group, and we found that tSVF was more effective in inhibiting cavernous fibrosis, restoring the content in the penis of hypertensive rats. Moreover, the expression of TGF‐*β*2, TNF‐*α*, and IL‐1*β* in the tSVF group was also lower than that in the cSVF group. Consequently, the expression of EndMT markers in tSVF group was closer to that in the control group. In order to figure out the mechanism by which cSVF and tSVF function in vivo, we previously labeled cSVF and tSVF with CM‐Dil and continuously traced the two fractions after the injection into rats to assess their longevity and differentiation capacity. Our in vivo tracking results of tSVF revealed a larger CM‐Dil–labeled area within the cavernous tissue compared to conventional stem cell therapies. Moreover, over time, these labeled structures not only exhibited morphology closely resembling the native tissue architecture but also demonstrated functional characteristics of endothelial cells, such as the secretion of eNOS. Based on these observations, we propose the following interpretations: First, the CM‐Dil labeling may have stained both cells and the inherent stromal components of tSVF, resulting in initially higher fluorescence signals in the tSVF group compared to cSVF. Although the CM‐Dil signal gradually decreased over time due to local blood flow and metabolic clearance, observable CM‐Dil–positive areas persisted in the tSVF group, unlike in the cSVF group where the signal nearly vanished. These residual structures in the tSVF group showed high structural and functional integration with the host tissue. This may be attributed to the ECM components in tSVF, which could support the differentiation of functional cells (e.g., endothelial cells or adipose‐derived mesenchymal stem cells) into endothelial lineages or directly facilitate endothelial repair under inflammatory stimulation. Cell functions, such as anchorage, morphogenesis, signaling, and survival, can be affected by the composition and mechanical properties of ECM [75]. tSVF is an exciting new prospect because—unlike traditional cSVF—it keeps the ECM intact, offers a growth scaffold for cells, and provides a buffer zone for extracellular secretion [76]. Immunofluorescence costaining of CM‐Dil and PCNA indicated pronounced proliferative activity in host cells adjacent to the CM‐Dil‐positive areas. This suggests that both cSVF and tSVF may modulate the pathophysiological responses induced by L‐NAME via paracrine signaling mechanisms, thereby promoting tissue regeneration. In our in vitro experiments, tSVF significantly inhibited EndMT even in the absence of direct contact with endothelial cells, supporting the notion that its therapeutic effects are mediated through paracrine mechanisms. Finally, although every effort was made to minimize potential confounding factors, we cannot entirely rule out the possibility that phagocytosis of the dye by host cells may have influenced the interpretation of the tracking data. This could potentially lead to an overestimation of tSVF’s retention and regenerative contribution.

In addition, compared with cSVF, tSVF has another advantage that tSVF production does not involve enzymatic hydrolysis, and the technique has advantages over cSVF in convenience, speed, and ease of clinical application [77]. The latter is particularly relevant given that some countries restrict the use of certain hydrolytic enzymes for clinical application [78, 79].

Although our findings provided evidence that tSVF is effective for treating hypertensive ED, this study still remained some limitations. We reviewed only the effects of cSVF and tSVF on the corpus cavernosum, ignoring the potential risk of embolism and tumorigenesis from the fractions entering other organs via blood circulation [80, 81]. Now that it has been established that tSVF can exert a therapeutic effect by proliferating and differentiating in vivo, the next step is to clarify how tSVF exerts its effects by secreting cytokines.

## 5. Conclusion

To the best of our knowledge, this study is the first to explore tSVF as a possible treatment for ED. We found that tSVF transplantation significantly recovered erectile function in a hypertensive rat model, restoring ICPmax/MAP and total ICP/MAP ratios. tSVF not only restored the endothelial content and lowered fibrosis but also suppressed inflammation‐induced EndMT both in vivo and vitro, which can be attributed to that tSVF suppresses EndMT by inhibiting the TGF‐*β*2–Smad2/Smad3 signaling pathway. Lastly, we demonstrated that tSVF is longevity and exhibits some self‐differentiation ability in vivo. In conclusion, tSVF is a promising therapeutic candidate for the treatment of hypertensive ED.

## Ethics Statement

The animal experiments were approved by the Institutional Animal Care and Use Committee of Nanjing Medical University.

## Consent

The authors have nothing to report.

## Conflicts of Interest

The authors declare no conflicts of interest.

## Author Contributions

**Cheng Shao**: investigation, validation, data curation, writing – original draft preparation. **Yi Sun**: data curation, formal analysis, validation. **Jun Zhao**: visualization, investigation, software. **Chao Ju**: resources. **Tianli Yang**: software. **Jingyu Liu**: validation. **Liuhua Zhou**: writing – review and editing. **Feng Zhao**: methodology, resources, supervision. **Ruipeng Jia**: conceptualization, resources, funding acquisition, project administration, supervision.

## Funding

This work was supported by the National Natural Science Foundation of China Grants (92049111 and 81902602).

## Data Availability

The data that support the findings of this study are available from the corresponding author upon reasonable request.

## References

[1] Salonia A. , Bettocchi C. , and Boeri L. , et al.European Association of Urology Guidelines on Sexual and Reproductive Health—2021 Update: Male Sexual Dysfunction, European Urology. (2021) 80, no. 3, 333–357, 10.1016/j.eururo.2021.06.007. 34183196

[2] McMahon C. G. , Current Diagnosis and Management of Erectile Dysfunction, Medical Journal of Australia. (2019) 210, no. 10, 469–476, 10.5694/mja2.50167, 2-s2.0-85066022558. 31099420

[3] Naya Y. , Mizutani Y. , and Ochiai A. , et al.Preliminary Report of Association of Chronic Diseases and Erectile Dysfunction in Middle-Aged Men in Japan, Urology. (2003) 62, 532–536. 12946762 10.1016/s0090-4295(03)00383-2

[4] Kang S. Y. , Lee J. A. , and Sunwoo S. , et al.Prevalence of Sexual Dysfunction and Associated Risk Factors in Middle-Aged and Elderly Korean Men in Primary Care, The Journal of Sex Research. (2016) 53, no. 9, 1165–1178, 10.1080/00224499.2016.1174657, 2-s2.0-84969832815. 27215144

[5] De Oliveira A. A. and Nunes K. P. , Hypertension and Erectile Dysfunction: Breaking Down the Challenges Am, Journal of Hypertension. (2021) 34, 134–142. 10.1093/ajh/hpaa143 32866225

[6] Papatsoris A. G. and Korantzopoulos P. G. , Hypertension, Antihypertensive Therapy, and Erectile Dysfunction, Angiology, 2006, 57, no. 1, 47–52, 10.1177/000331970605700107, 2-s2.0-33645300782. 16444456

[7] Zimmerlin L. , Donnenberg V. S. , and Pfeifer M. E. , et al.Stromal Vascular Progenitors in Adult Human Adipose Tissue, Cytometry Part A: The Journal of the International Society for Analytical Cytology. (2010) 77, 22–30. 19852056 10.1002/cyto.a.20813 PMC4148047

[8] Yang T. , Zhao F. , and Zhao J. , et al.Negatively Charged Bladder Acellular Matrix Loaded With Positively Charged Adipose-Derived Mesenchymal Stem Cell-Derived Small Extracellular Vesicles for Bladder Tissue Engineering, Journal of Controlled Release. (2023) 364, 718–733, 10.1016/j.jconrel.2023.10.048. 37944669

[9] Zhao F. , Zhou L. , and Liu J. , et al.Construction of a Vascularized Bladder With Autologous Adipose-Derived Stromal Vascular Fraction Cells Combined With Bladder Acellular Matrix via Tissue Engineering, Journal of Tissue Engineering. (2019) 10, 10.1177/2041731419891256. PMC6886281 31827758

[10] Trivisonno A. , Alexander R. W. , and Baldari S. , et al.Intraoperative Strategies for Minimal Manipulation of Autologous Adipose Tissue for Cell- and Tissue-Based Therapies: Concise Review, Stem Cells Translational Medicine. (2019) 8, no. 12, 1265–1271, 10.1002/sctm.19-0166. 31599497 PMC6877766

[11] Condé-Green A. , Kotamarti V. S. , and Sherman L. S. , et al.Shift toward Mechanical Isolation of Adipose-Derived Stromal Vascular Fraction: Review of Upcoming Techniques, Plastic and Reconstructive Surgery – Global Open. (2016) 4, e1017. 27757339 10.1097/GOX.0000000000001017 PMC5055005

[12] Baldari S. , Di Rocco G. , Piccoli M. , Pozzobon M. , Muraca M. , and Toietta G. , Challenges and Strategies for Improving the Regenerative Effects of Mesenchymal Stromal Cell-Based Therapies, International Journal of Molecular Sciences, 2017, 18, no. 10, 2087 10.3390/ijms18102087, 2-s2.0-85030715354, 2087. PMC5666769 28974046

[13] Alexander R. W. , Biocellular Regenerative Medicine: Use of Adipose-Derived Stem/Stromal Cells and It’s Native Bioactive Matrix, Physical Medicine and Rehabilitation Clinics of North America. (2016) 27, 871–891. 27788905 10.1016/j.pmr.2016.06.005

[14] Song K. , Jin H. , and Park J. , et al.Intracavernous Delivery of Stromal Vascular Fraction Restores Erectile Function Through Production of Angiogenic Factors in a Mouse Model of Cavernous Nerve Injury, The Journal of Sexual Medicine. (2014) 11, no. 8, 1962–1973, 10.1111/jsm.12597, 2-s2.0-84905013261. 24902866

[15] Qiu X. , Fandel T. M. , and Ferretti L. , et al.Both Immediate and Delayed Intracavernous Injection of Autologous Adipose-Derived Stromal Vascular Fraction Enhances Recovery of Erectile Function in a Rat Model of Cavernous Nerve Injury, European Urology. (2012) 62, no. 4, 720–727, 10.1016/j.eururo.2012.02.003, 2-s2.0-84865679476. 22397847 PMC3542983

[16] You D. , Jang M. J. , and Kim B. H. , et al.Comparative Study of Autologous Stromal Vascular Fraction and Adipose-Derived Stem Cells for Erectile Function Recovery in a Rat Model of Cavernous Nerve Injury Stem Cells, Journal of Translational Medicine. (2015) 4, 351–358. 10.5966/sctm.2014-0161 PMC4367505 25792486

[17] Ghosh A. K. , Quaggin S. E. , and Vaughan D. E. , Molecular Basis of Organ Fibrosis: Potential Therapeutic Approaches, Experimental Biology and Medicine. (2013) 238, no. 5, 461–481, 10.1177/1535370213489441, 2-s2.0-84881577297. 23856899

[18] Potenta S. , Zeisberg E. , and Kalluri R. , The Role of Endothelial-to-Mesenchymal Transition in Cancer Progression, British Journal of Cancer. (2008) 99, no. 9, 1375–1379, 10.1038/sj.bjc.6604662, 2-s2.0-55249126800. 18797460 PMC2579683

[19] Gorelova A. , Berman M. , and Al Ghouleh I. , Endothelial-to-Mesenchymal Transition in Pulmonary Arterial Hypertension, Antioxidants & Redox Signaling. (2021) 34, no. 12, 891–914, 10.1089/ars.2020.8169. 32746619 PMC8035923

[20] Helmke A. , Casper J. , and Nordlohne J. , et al.Endothelial-to-Mesenchymal Transition Shapes the Atherosclerotic Plaque and Modulates Macrophage Function, The FASEB Journal. (2019) 33, 2278–2289. 30260706 10.1096/fj.201801238R

[21] Bischoff J. , Endothelial-to-Mesenchymal Transition, Circulation Research. (2019) 124, 1163–1165. 30973806 10.1161/CIRCRESAHA.119.314813 PMC6540806

[22] Yoshimatsu Y. , Wakabayashi I. , and Kimuro S. , et al.TNF-*α* Enhances TGF-*β*-Induced Endothelial-to-Mesenchymal Transition via TGF-*β* Signal Augmentation, Cancer Science. (2020) 111, 2385–2399. 32385953 10.1111/cas.14455 PMC7385392

[23] Takagaki Y. , Lee S. M. , Dongqing Z. , Kitada M. , Kanasaki K. , and Koya D. , Endothelial Autophagy Deficiency Induces IL6 - Dependent Endothelial Mesenchymal Transition and Organ Fibrosis., Autophagy. (2020) 16, no. 10, 1905–1914, 10.1080/15548627.2020.1713641. 31965901 PMC8386622

[24] Zhang H. , Feng C. , and He S. , et al.Leech-Centipede Granules Suppress EndMT to Improve Erectile Dysfunction in Rats With Diabetes Mellitus via TGF-*β*/Smad Pathway, Journal of Chinese Integrative Medicine. (2023) 29, 28–36. 10.1007/s11655-022-3728-z 36542225

[25] Zhou L. , Song K. , and Xu L. , et al.Protective Effects of Uncultured Adipose-Derived Stromal Vascular Fraction on Testicular Injury Induced by Torsion-Detorsion in Rats Stem Cells, Journal of Translational Medicine. (2018) 8, 383–391. 10.1002/sctm.18-0063 PMC6431687 30569668

[26] Kopincová J. , Púzserová A. , and Bernátová I. , L-NAME in the Cardiovascular System - Nitric Oxide Synthase Activator?, Pharmacological Reports. (2012) 64, no. 3, 511–520, 10.1016/S1734-1140(12)70846-0, 2-s2.0-84866118438. 22814004

[27] Gur S. , Kadowitz P. J. , and Gurkan L. , et al.Chronic Inhibition of Nitric-Oxide Synthase Induces Hypertension and Erectile Dysfunction in the Rat That Is Not Reversed by Sildenafil, BJU International. (2010) 106, no. 1, 78–83, 10.1111/j.1464-410X.2009.09104.x, 2-s2.0-77953211239. 20002674

[28] Su-Hong C. , Qi C. , Bo L. , Jian-Li G. , Jie S. , and Gui-Yuan L. , Antihypertensive Effect of Radix Paeoniae Alba in Spontaneously Hypertensive Rats and Excessive Alcohol Intake and High Fat Diet Induced Hypertensive Rats, Evidence-Based Complementary and Alternative Medicine. (2015) 8, 10.1155/2015/731237, 2-s2.0-84924279667, 731237. PMC4345252 25784949

[29] Krishnamoorthi M. K. , Thandavarayan R. A. , Youker K. A. , and Bhimaraj A. , An In Vitro Platform to Study Reversible Endothelial-to-Mesenchymal Transition, Frontiers in Pharmacology. (2022) 13, 912660. 35814231 10.3389/fphar.2022.912660 PMC9259860

[30] Silva F. C. , Araújo B. J. , and Cordeiro C.S , et al.Endothelial Dysfunction Due to the Inhibition of the Synthesis of Nitric Oxide: Proposal and Characterization of an In Vitro Cellular Model, Frontiers in Physiology. (2022) 13, 978378. 36467706 10.3389/fphys.2022.978378 PMC9714775

[31] Wang L. , Cheng C. K. , Yi M. , Lui K. O. , and Huang Y. , Targeting Endothelial Dysfunction and Inflammation, Journal of Molecular and Cellular Cardiology. (2022) 168, 58–67, 10.1016/j.yjmcc.2022.04.011. 35460762

[32] Intengan H. D. and Schiffrin E. L. , Vascular Remodeling in Hypertension: Roles of Apoptosis, Inflammation, and Fibrosis Hypertens, Dallas Tex. (2001) 38, 581–587. 10.1161/hy09t1.096249 11566935

[33] Frohlich E. D. , Fibrosis and Ischemia: The Real Risks in Hypertensive Heart Disease, Journal of Hypertension. (2001) 14, 194S–199S. 10.1016/s0895-7061(01)02088-x 11411756

[34] Nunes K. P. , Labazi H. , and Webb R. C. , New Insights into Hypertension-Associated Erectile Dysfunction, Current Opinion in Nephrology and Hypertension. (2012) 21, no. 2, 163–170, 10.1097/MNH.0b013e32835021bd, 2-s2.0-84857043055. 22240443 PMC4004343

[35] Dean R. C. and Lue T. F. , Physiology of Penile Erection and Pathophysiology of Erectile Dysfunction, Urologic Clinics of North America. (2005) 32, 379–395. 16291031 10.1016/j.ucl.2005.08.007 PMC1351051

[36] Ferraz M. M. D. , Quintella S. L. , Parcial A. L. N. , and Ferraz M. R. , The Effects of Sildenafil After Chronic L-NAME Administration in Male Rat Sexual Behavior, Pharmacology Biochemistry and Behavior. (2016) 15, 146–147. 10.1016/j.pbb.2016.04.004 27132237

[37] Ferraz M. R. , Ferraz M. M. D. , Santos R. , and Soares de Moura R. , Preventing L-NAME Inhibitory Effects on Rat Sexual Behavior With Hydralazine, Isradipine or Captopril Co-Treatment, Pharmacology Biochemistry and Behavior. (2003) 75, no. 2, 265–272, 10.1016/S0091-3057(03)00077-7, 2-s2.0-0038784683. 12873615

[38] Ademosun A. O. , Mohammed A. , Oboh G. , and Ajeigbe O. F. , Influence of Lemon (*Citrus limon*) and Lime (*Citrus aurantifolia*) Juices on the Erectogenic Properties of Sildenafil in Rats With L-NAME-Induced Erectile Dysfunction, Journal of Food Biochemistry. (2022) 46, e14074. 35034363 10.1111/jfbc.14074

[39] Yang M. , Chen X. , and Zhang M. , et al.hUC-MSC Preserves Erectile Function by Restoring Mitochondrial Mass of Penile Smooth Muscle Cells in a Rat Model of Cavernous Nerve Injury via SIRT1/PGC-1a/TFAM Signaling, Biological Research. (2025) 58, no. 1, 10.1186/s40659-024-00578-y. PMC11773750 39871297

[40] Hong Y. , Feng Z. , and Ge Y. , et al.MiR-145-Enriched BMSCs-Derived Exosomes Ameliorate Neurogenic Erectile Dysfunction in Aged Rats via TGFBR2 Inhibition, Regenerative Therapy. (2025) 29, 455–465. 40308644 10.1016/j.reth.2025.04.004 PMC12041780

[41] Zhang X. , Yang M. , Chen X. , Zhang M. , Peng Y. , and Lu M. , Melatonin-Pretreated Mesenchymal Stem Cell-Derived Exosomes Alleviate Cavernous Fibrosis in a Rat Model of Nerve Injury-Induced Erectile Dysfunction via miR-145-5p/TGF-*β*/Smad Axis, Stem Cell Research & Therapy. (2025) 16, no. 1, 10.1186/s13287-025-04173-0, 96. 40001250 PMC11863846

[42] Sun C. , Lin H. , and Yu W. , et al.Neurotrophic Effect of Bone Marrow Mesenchymal Stem Cells for Erectile Dysfunction in Diabetic Rats: MSCs for Diabetes-Associated ED, Journal of Andrology. (2012) 35, 601–607. 10.1111/j.1365-2605.2012.01250.x 22428616

[43] Kim S. W. , Zhu G. Q. , and Bae W. J. , Mesenchymal Stem Cells Treatment for Erectile Dysfunction in Diabetic Rats Sex, Medical Reviews International. (2020) 8, 114–121. 10.1016/j.sxmr.2019.09.003 31653438

[44] Chen Z. , Han X. , Ouyang X. , Fang J. , Huang X. , and Wei H. , Transplantation of Induced Pluripotent Stem Cell-Derived Mesenchymal Stem Cells Improved Erectile Dysfunction Induced by Cavernous Nerve Injury, Theranostics. (2019) 9, no. 22, 6354–6368, 10.7150/thno.34008, 2-s2.0-85071834100. 31588222 PMC6771238

[45] Pérez-Aizpurua X. , Garranzo-Ibarrola M. , and Simón-Rodríguez C. , et al.Stem Cell Therapy for Erectile Dysfunction: A Step towards a Future Treatment, Life, 2023, 13, 502. 10.3390/life13020502 PMC9963846 36836859

[46] Zhou F. , Hui Y. , and Xu Y. , et al.Effects of Adipose-Derived Stem Cells Plus Insulin on Erectile Function in Streptozotocin-Induced Diabetic Rats, International Urology and Nephrology. (2016) 48, no. 5, 657–669, 10.1007/s11255-016-1221-3, 2-s2.0-84955572188. 26820518

[47] Garcia M. , Fandel T. , and Lin G. , et al.Treatment of Erectile Dysfunction in the Obese Type 2 Diabetic ZDF Rat With Adipose Tissue-Derived Stem Cells, The Journal of Sexual Medicine. (2010) 7, no. 1, 89–98, 10.1111/j.1743-6109.2009.01541.x, 2-s2.0-74049113244. 20104670 PMC2904063

[48] Vargel I. , Tuncel A. , Baysal N. , Hartuç-Çevik İ , and Korkusuz F. , Autologous Adipose-Derived Tissue Stromal Vascular Fraction (AD-tSVF) for Knee Osteoarthritis, International Journal of Molecular Sciences. (2022) 23, no. 21, 10.3390/ijms232113517, 13517. 36362308 PMC9658499

[49] van Boxtel J. , Vonk L. A. , Stevens H. P. , and van Dongen J. A. , Mechanically Derived Tissue Stromal Vascular Fraction Acts Anti-Inflammatory on TNF Alpha-Stimulated Chondrocytes In Vitro, Bioengineering. (2022) 9, 345. 35892757 10.3390/bioengineering9080345 PMC9332748

[50] Chen W. , He Z. , and Li S. , et al.The Effect of Tissue Stromal Vascular Fraction as Compared to Cellular Stromal Vascular Fraction to Treat Anal Sphincter Incontinence, Bioengineering. (2022) 10, 32. 36671604 10.3390/bioengineering10010032 PMC9854502

[51] van Dongen J. , Harmsen M. , van der Lei B. , and Stevens H. , Augmentation of Dermal Wound Healing by Adipose Tissue-Derived Stromal Cells (ASC), Bioengineering. (2018) 5, 91. 30373121 10.3390/bioengineering5040091 PMC6316823

[52] Zou H. , Zhang X. , and Chen W. , et al.Vascular Endothelium Is the Basic Way for Stem Cells to Treat Erectile Dysfunction: A Bibliometric Study, Cell Death Discovery. (2023) 9, 143. 37127677 10.1038/s41420-023-01443-9 PMC10151332

[53] Xu Y. and Kovacic J. C. , Endothelial to Mesenchymal Transition in Health and Disease, Annual Review of Physiology. (2023) 85, no. 1, 245–267, 10.1146/annurev-physiol-032222-080806. 36266259

[54] Alvandi Z. and Bischoff J. , Endothelial-Mesenchymal Transition in Cardiovascular Disease, Arteriosclerosis, Thrombosis, and Vascular Biology. (2021) 41, no. 9, 2357–2369, 10.1161/ATVBAHA.121.313788. 34196216 PMC8387428

[55] Jackson A. O. , Zhang J. , Jiang Z. , and Yin K. , Endothelial-to-Mesenchymal Transition: A Novel Therapeutic Target for Cardiovascular Diseases, Trends in Cardiovascular Medicine. (2017) 27, 383–393. 28438397 10.1016/j.tcm.2017.03.003

[56] Burchardt T. , Burchardt M. , and Karden J. , et al.Reduction of Endothelial and Smooth Muscle Density in the Corpora Cavernosa of the Streptozotocin Induced Diabetic Rat, Journal of Urology. (2000) 164, no. 5, 1807–1811, 10.1016/S0022-5347(05)67111-X, 2-s2.0-0033782371. 11025774

[57] Shen B. , Gu T. , and Shen Z. , et al. *Escherichia coli* Promotes Endothelial to Mesenchymal Transformation of Liver Sinusoidal Endothelial Cells and Exacerbates Nonalcoholic Fatty Liver Disease Via Its Flagellin, Cellular and Molecular Gastroenterology and Hepatology. (2023) 16, no. 6, 857–879, 10.1016/j.jcmgh.2023.08.001. 37572735 PMC10598062

[58] Cheng D. , Lian W. , and Jia X. , et al.LGALS3 Regulates Endothelial-to-Mesenchymal Transition via PI3K/AKT Signaling Pathway in Silica-Induced Pulmonary Fibrosis, Toxicology. (2024) 509, 153962. 39353502 10.1016/j.tox.2024.153962

[59] Huang J. , Liu Y. , and Shi M. , et al.Empagliflozin Attenuating Renal Interstitial Fibrosis in Diabetic Kidney Disease by Inhibiting Lymphangiogenesis and Lymphatic Endothelial-to-Mesenchymal Transition via the VEGF-C/VEGFR3 Pathway, Biomedicine & Pharmacotherapy. (2024) 180, 117589. 39418962 10.1016/j.biopha.2024.117589

[60] Lai Y.-J. , Tsai F.-C. , and Chang G.-J. , et al.MiR-181b Targets Semaphorin 3A to Mediate TGF-*β*–Induced Endothelial-Mesenchymal Transition Related to Atrial Fibrillation, Journal of Clinical Investigation. (2022) 132, e142548. 35775491 10.1172/JCI142548 PMC9246393

[61] Goumans M.-J. , van Zonneveld A. J. , and ten Dijke P. , Transforming Growth Factor Beta-Induced Endothelial-to-Mesenchymal Transition: A Switch to Cardiac Fibrosis?, Trends in Cardiovascular Medicine. (2008) 18, 293–298. 19345316 10.1016/j.tcm.2009.01.001

[62] van Meeteren L. A. and ten Dijke P. , Regulation of Endothelial Cell Plasticity by TGF-*β* Cell Tissue Res, Cell and Tissue Research, 2012, 347, 177–186. 10.1007/s00441-011-1222-6 PMC3250609 21866313

[63] Chen P.-Y. , Qin L. , and Baeyens N. , et al.Endothelial-to-Mesenchymal Transition Drives Atherosclerosis Progression, Journal of Clinical Investigation. (2015) 125, no. 12, 4514–4528, 10.1172/JCI82719, 2-s2.0-84948808417. 26517696 PMC4665771

[64] Pérez L. , Muñoz-Durango N. , and Riedel C. A. , et al.Endothelial-to-Mesenchymal Transition: Cytokine-Mediated Pathways That Determine Endothelial Fibrosis Under Inflammatory Conditions, Cytokine & Growth Factor Reviews. (2017) 33, 41–54, 10.1016/j.cytogfr.2016.09.002, 2-s2.0-84998980152. 27692608

[65] Arciniegas E. , Sutton A. B. , Allen T. D. , and Schor A. M. , Transforming Growth Factor Beta 1 Promotes the Differentiation of Endothelial Cells Into Smooth Muscle-Like Cells In Vitro, Journal of Cell Science. (1992) 103, no. 2, 521–529, 10.1242/jcs.103.2.521. 1478952

[66] Zhang J. , Zhang Z. , Zhang D. Y. , Zhu J. , Zhang T. , and Wang C. , MicroRNA. 126 Inhibits the Transition of Endothelial Progenitor Cells to Mesenchymal Cells via the PIK3R2-PI3K/Akt Signalling Pathway, PloS ONE, 2013, 8, e83294. 10.1371/journal.pone.0083294 PMC3862723 24349482

[67] Medici D. , Potenta S. , and Kalluri R. , Transforming Growth Factor-*β*2 Promotes Snail-Mediated Endothelial-Mesenchymal Transition Through Convergence of Smad-Dependent and Smad-Independent Signalling, Biochemical Journal. (2011) 437, 515–520. 21585337 10.1042/BJ20101500 PMC4457510

[68] Ghosh A. K. , Nagpal V. , Covington J. W. , Michaels M. A. , and Vaughan D. E. , Molecular Basis of Cardiac Endothelial-to-Mesenchymal Transition (EndMT): Differential Expression of microRNAs During EndMT, Cellular Signalling. (2012) 24, no. 5, 1031–1036, 10.1016/j.cellsig.2011.12.024, 2-s2.0-84857111170. 22245495 PMC3298765

[69] Sabbineni H. , Verma A. , and Somanath P. R. , Isoform-Specific Effects of Transforming Growth Factor *β* on Endothelial-to-Mesenchymal Transition, Journal of Cellular Physiology. (2018) 233, no. 11, 8418–8428, 10.1002/jcp.26801, 2-s2.0-85054054891. 29856065 PMC6415927

[70] Romero L. I. , Zhang D.-N. , Herron G. S. , and Karasek M. A. , Interleukin-1 Induces Major Phenotypic Changes in Human Skin Microvascular Endothelial Cells, Journal of Cellular Physiology. (1997) 173, no. 1, 84–92. 9326452 10.1002/(SICI)1097-4652(199710)173:1<84::AID-JCP10>3.0.CO;2-N

[71] Chaudhuri V. , Zhou L. , and Karasek M. , Inflammatory Cytokines Induce the Transformation of Human Dermal Microvascular Endothelial Cells Into Myofibroblasts: A Potential Role in Skin Fibrogenesis, Journal of Cutaneous Pathology. (2007) 34, no. 2, 146–153, 10.1111/j.1600-0560.2006.00584.x, 2-s2.0-33846409095. 17244026

[72] Maleszewska M. , Moonen J. R. , Huijkman N. , van de Sluis B. , Krenning G. , and Harmsen M. C. , IL-1*β* and TGF*β*2 Synergistically Induce Endothelial to Mesenchymal Transition in an NF*κ*B-Dependent Manner Immunobiology, 2013, 218, 443–454. 10.1016/j.imbio.2012.05.026 22739237

[73] Rieder F. , Kessler S. P. , and West G. A. , et al.Inflammation-Induced Endothelial-to-Mesenchymal Transition: A Novel Mechanism of Intestinal Fibrosis, Journal of Pathology. (2011) 179, 2660–2673. 10.1016/j.ajpath.2011.07.042 PMC3204019 21945322

[74] Mahler G. J. , Farrar E. J. , and Butcher J. T. , Inflammatory Cytokines Promote Mesenchymal Transformation in Embryonic and Adult Valve Endothelial Cells, Arteriosclerosis, Thrombosis, and Vascular Biology. (2013) 33, no. 1, 121–130, 10.1161/ATVBAHA.112.300504, 2-s2.0-84871737124. 23104848 PMC3694265

[75] Valdoz J. C. , Johnson B. C. , and Jacobs D. J. , et al.The ECM: To Scaffold, or Not to Scaffold, That Is the Question, International Journal of Molecular Sciences. (2021) 22, no. 23, 10.3390/ijms222312690, 12690. 34884495 PMC8657545

[76] Dongen J. A. , Getova V. , and Brouwer L. A. , et al.Adipose Tissue-Derived Extracellular Matrix Hydrogels as a Release Platform for Secreted Paracrine Factors, Journal of Tissue Engineering and Regenerative Medicine. (2019) 13, no. 6, 973–985, 10.1002/term.2843, 2-s2.0-85064501457. 30808068 PMC6593768

[77] van Dongen J. A. , Tuin A. J. , Spiekman M. , Jansma J. , van der Lei B. , and Harmsen M. C. , Comparison of Intraoperative Procedures for Isolation of Clinical Grade Stromal Vascular Fraction for Regenerative Purposes: A Systematic Review, Journal of Tissue Engineering and Regenerative Medicine. (2018) 12, no. 1, e261–e274, 10.1002/term.2407, 2-s2.0-85020916631. 28084666

[78] Chaput B. , Bertheuil N. , and Escubes M. , et al.Mechanically Isolated Stromal Vascular Fraction Provides a Valid and Useful Collagenase-Free Alternative Technique: A Comparative Study, Plastic & Reconstructive Surgery. (2016) 138, no. 4, 807–819, 10.1097/PRS.0000000000002494, 2-s2.0-84974830979. 27307342

[79] Aronowitz J. A. , Lockhart R. A. , and Hakakian C. S. , Mechanical Versus Enzymatic Isolation of Stromal Vascular Fraction Cells from Adipose Tissue, SpringerPlus. (2015) 4, 713. 26636001 10.1186/s40064-015-1509-2 PMC4656256

[80] Xu Y. , Yang Y. , and Zheng H. , et al.Intracavernous Injection of Size-Specific Stem Cell Spheroids for Neurogenic Erectile Dysfunction: Efficacy and Risk Versus Single Cells, eBioMedicine. (2020) 52, 102656. 32062355 10.1016/j.ebiom.2020.102656 PMC7016386

[81] Yu J. M. , Jun E. S. , Bae Y. C. , and Jung J. S. , Mesenchymal Stem Cells Derived From Human Adipose Tissues Favor Tumor Cell Growth In Vivo, Stem Cells and Development. (2008) 17, 463–473. 18522494 10.1089/scd.2007.0181

